# Modulation of diabetes-related retinal pathophysiology by PTX3

**DOI:** 10.1073/pnas.2320034121

**Published:** 2024-09-30

**Authors:** Varun Pathak, Pietro M. Bertelli, Edoardo Pedrini, Kevin Harkin, Elisa Peixoto, Lynsey-Dawn Allen, Kiran Mcloughlin, Natasha D. Chavda, Kevin J. Hamill, Jasenka Guduric-Fuchs, Antonio Inforzato, Barbara Bottazzi, Alan W. Stitt, Reinhold J. Medina

**Affiliations:** ^a^The Wellcome-Wolfson Institute for Experimental Medicine, Queen’s University Belfast, Belfast BT9 7BL, United Kingdom; ^b^Department for Eye and Vision Sciences, Institute of Life Course and Medical Sciences, University of Liverpool, Liverpool L7 8TX, United Kingdom; ^c^Laboratory of Cellular and Humoral Innate Immunity, Istituto di Ricovero e Cura a Carattere Scientifico Humanitas Research Hospital, Rozzano, Milan 20089, Italy; ^d^Department of Biomedical Sciences, Humanitas University, Milan 20072, Italy

**Keywords:** retinopathy, diabetic retinopathy, PTX3, gliosis, microglia

## Abstract

Diabetic retinopathy (DR) is a common complication of diabetes characterized by vascular pathology and neuroinflammation. Pentraxin 3 (PTX3) is a soluble pattern recognition molecule that functions at the crossroads between innate immunity, inflammation, and tissue remodeling. DR is known to involve inflammatory pathways, although the potential relevance of PTX3 has not been explored. We found that PTX3 protein levels increased in the retina of diabetic mice. Similarly, evaluation of a publicly available transcriptomic human dataset revealed increased PTX3 expression in DR with diabetic macular edema and proliferative retinopathy, when compared to nondiabetic retinas or diabetic retinas without complications. To further understand the role of PTX3 within DR, we employed the streptozotocin-induced diabetes model in PTX3 knockout mice (PTX3^KO^), which were followed up for 9 mo to evaluate hallmarks of disease progression. In diabetic PTX3^KO^ mice, we observed decreased reactive gliosis, diminished microglia activation, and reduced vasodegeneration, when compared to diabetic PTX3 wild-type littermates. The decrease in DR–associated pathological features in PTX3^KO^ retinas translated into preserved visual function, as evidenced by improved optokinetic response, restored b-wave amplitude in electroretinograms, and attenuated neurodegeneration. We showed that PTX3 induced an inflammatory phenotype in human retinal macroglia, characterized by GFAP upregulation and increased secretion of IL6 and PAI-1. We confirmed that PTX3 was required for TNF-α-induced reactive gliosis, as PTX3^KO^ retinal explants did not up-regulate GFAP in response to TNF-α. This study reveals a unique role for PTX3 as an enhancer of sterile inflammation in DR, which drives pathogenesis and ultimately visual impairment.

## Significance

Diabetic retinopathy (DR) is a leading cause of blindness, and its pathogenesis involves inflammation. This study identifies a role for Pentraxin 3 (PTX3) in driving various pathological features in the diabetic retina. Our findings revealed that PTX3-deficient mice were protected from visual impairment induced by diabetes. Diabetic PTX3-knockout retinas showed attenuated DR hallmarks, including reactive gliosis, microglia activation, and vasoneurodegeneration. We found that PTX3 was required for TNF-induced retinal inflammation, inducing GFAP upregulation and IL6 and PAI-1 secretion in retinal macroglia. This work proposes PTX3 as a molecular driver of sterile inflammation in the diabetic retina.

Diabetes mellitus is a global health challenge with a prevalence projected to reach 592 million people by 2035 (1). Diabetic retinopathy (DR) is the most frequently occurring diabetic complication, and it remains a leading cause of visual impairment being responsible for 2.6% of blindness cases worldwide (2). The pathogenesis of DR involves multiple concurrent cellular processes, such as vascular degeneration, blood–retinal barrier disruption, hypoxia, glial dysfunction, immune cell activation, and neurodegeneration (3). Although sterile inflammation has been proposed as a driver of DR progression through retinal microglial activation and the release of cytokines like TNF-α, IL-1β, and IL6 (4), most therapeutic efforts have focused on blocking pathological angiogenesis in the late stage of proliferative DR by the use of pan-retinal laser photocoagulation or therapeutics targeting ischemia-linked secretion of VEGF. Furthermore, inflammatory factors sICAM-1, MCP1, and Pentraxin 3 (PTX3) are increased in the vitreous of patients with diabetic macular edema (DME) (5), and steroids remain an effective alternative treatment to anti-VEGFs (6).

Evolutionary related to C-reactive protein (CRP) and serum amyloid P component (SAP), the long pentraxin PTX3 is a soluble pattern recognition molecule (7), locally expressed by a number of immune and nonimmune cells at sites of infection (8), where it exerts host-protecting functions in the immune response to selected opportunistic pathogens (9). Besides its roles in innate immunity to infections, PTX3 has been reported to modulate sterile inflammation in preclinical models of myocardial infarction (10), intestinal ischemia (11), and acute lung injury (12). Accumulating evidence indicates that PTX3 is present in the human eye, where it is synthesized by different cell types, including retinal pigmented epithelial (RPE), endothelial, and myeloid cells (13). The locally made protein takes part in tissue homeostasis, for example, by dampening overactivation of the complement system (14), whose dysregulation is a hallmark of age-related macular degeneration (AMD), a neurodegenerative disease of the eye and a leading cause of vision loss in the elderly.

At present, little is known regarding the involvement of PTX3 in the pathogenesis of DR. Controversial are the findings on the association of PTX3 systemic (plasmatic) concentration with the disease status (15, 16). It appears, however, that PTX3 local levels in the aqueous humor of DR patients are higher than those in diabetic patients with no retinopathy or nondiabetic volunteers (17). This information notwithstanding, the biological relevance of PTX3 in DR remains unknown. In this study, we assessed the expression of PTX3 in the diabetic retina and investigated how PTX3 affects retinal cell biology during diabetes, using a 9-mo diabetes mouse model and human retinal cells in vitro. We provide evidence that PTX3 acts as an enhancer of Muller cell gliosis, microglial activation, and vaso-neurodegeneration. This is in line with our results showing that lack of PTX3 protected retinal function and vision in diabetic mice.

## Results

### PTX3 Accumulates in Murine DR and Is Highly Expressed in Human Diabetic Retinas with Complications.

**Fig. 1.**
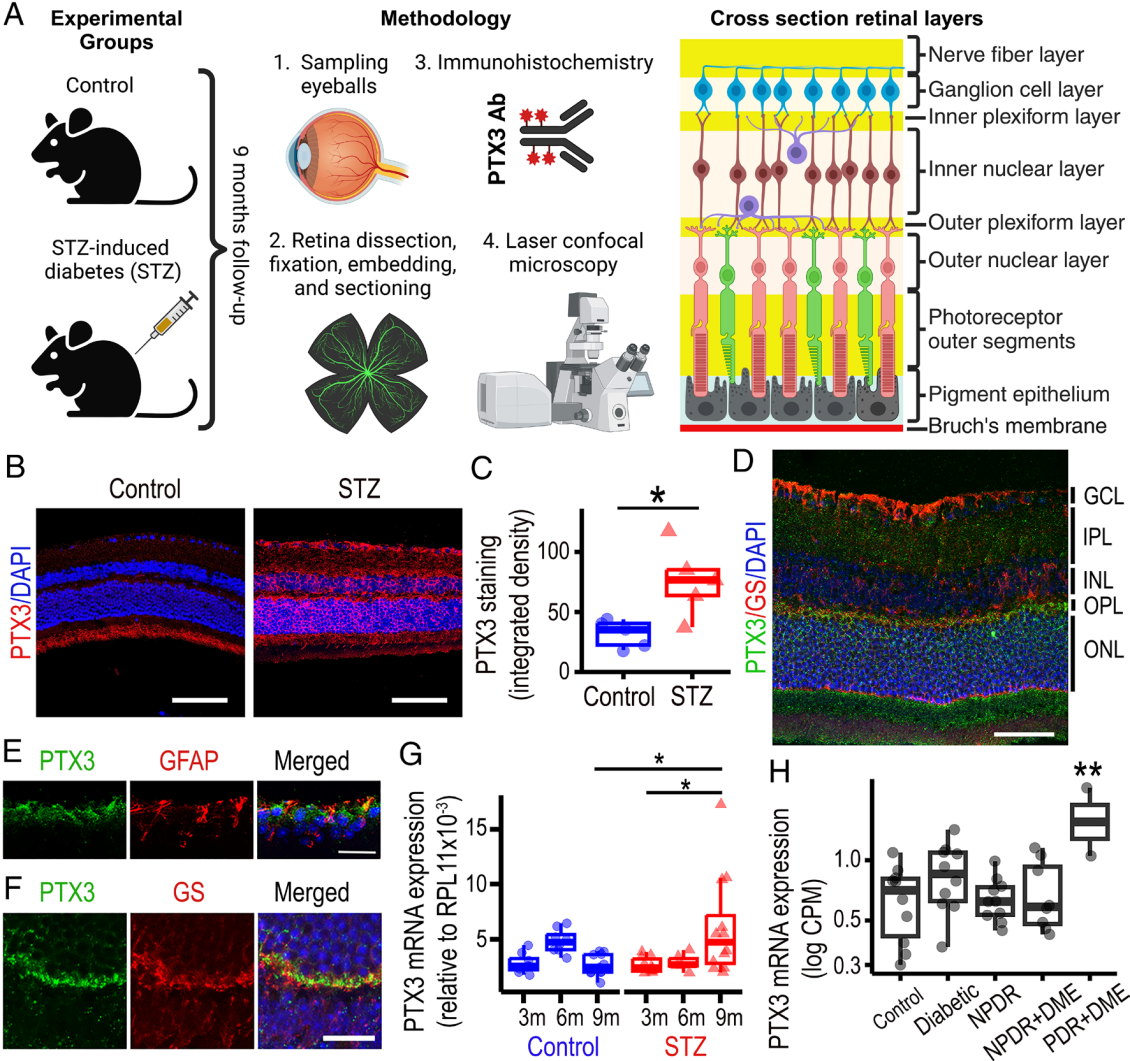
PTX3 is increased in diabetic retinas. (*A*) Experimental design, methods, and reference diagram for retinal cross-section identifying distinct cell types. Created with BioRender.com. (*B*) Immunohistochemistry for PTX3 (red) in cross-sections of peripheral retina in 9-mo diabetic mice (STZ) and age-matched controls. Nuclei counterstained with DAPI (blue). (Scale bar, 50 μm.) (*C*) Quantification of PTX3 staining area as integrated density, *P < 0.05, n = 5. (*D*) Immunostaining of mouse diabetic retinas with antibodies against PTX3 (green) and GS (red). (Scale bar, 50 μm.) (*E*) Fluorescent microscopy image of the nerve fiber and ganglion cell layers stained for PTX3 (green), GFAP (red), and DAPI (blue). (Scale bar, 25 μm.) (*F*) Representative image of the outer plexiform layer stained for PTX3 (green), GS (red), and DAPI (blue). (Scale bar, 20 μm.) (*G*) PTX3 gene expression evaluated by RT-qPCR in mouse whole retinal tissue at 3, 6, and 9 mo after STZ injection alongside controls. *P < 0.05. (*H*) PTX3 gene expression levels in human retinas across the natural history of diabetic retinopathy (DR) from GSE160306, **P < 0.01. NPDR: nonproliferative DR, PDR: proliferative DR, DME: diabetic macular edema.

We investigated the role of PTX3 in DR using the streptozotocin (STZ) type 1 diabetic mouse model and compared results with nondiabetic control mice. After 9 mo of diabetes, eyeballs were sampled, and retinas were processed for laser confocal microscopy after immunostaining with PTX3 antibody (Fig. 1*A*). We first assessed the expression and localization of the PTX3 protein in the peripheral and central retina of diabetic mice. Immunohistochemistry of peripheral retinal sections from the STZ-induced diabetic animals showed a significant increase in PTX3 protein staining at 9 mo from STZ administration, when compared to age-matched retinas from nondiabetic littermates (Fig. 1 *B* and *C*). This increase in PTX3 was not apparent at 3 mo and 6 mo of experimental diabetes (*SI Appendix*, Fig. S1*A*). Evaluation of central retinas showed similar results, with a significant increase in PTX3 protein expression at 9 mo after diabetes induction, but no difference found at 3 or 6 mo (*SI Appendix*, Fig. S1*B*). Retinas from the PTX3^KO^ mouse were used as negative controls and showed no staining for PTX3. Staining with only the 2ry antibody confirmed minimal background signal (*SI Appendix,* Fig. S2). High-resolution microscopy allowed PTX3 protein to be localized in the nerve fiber (NFL), outer plexiform (OPL), and outer nuclear (ONL) layers in wild-type retinas 9 mo after diabetes induction (Fig. 1*D*). Expression profiles for Glial Fibrillary Acidic Protein (GFAP) and Glutathione Synthase (GS), at the cellular level, did not colocalize with PTX3 (Fig. 1 *E* and *F*). Extracellular accumulation of PTX3 staining was evident in the NFL (Fig. 1*E*) and OPL (Fig. 1*F*). Considering the evidence that PTX3 incorporates into the extracellular matrix (ECM) (18), we performed colocalization experiments with a range of basement membrane-associated proteins. PTX3 immunoreactivity occurred in close association with Tenascin-C and Endostatin in the OPL, and with Versican and biotinylated hyaluronic acid binding protein in the inner limiting membrane (ILM) (*SI Appendix,* Fig. S3). PTX3 mRNA expression in whole murine retinal isolates showed a significant increase at 9-mo after diabetes induction, when compared to nondiabetic age-matched retinas (Fig. 1*G*). In addition, within the diabetic retinas, we observed a significantly increased PTX3 gene expression when comparing 3 mo to 9 mo after STZ injection (Fig. 1*G*). We then assessed GEO publicly available transcriptomics data from human DR and control samples. This published study (19), following informed consent, obtained human eyes from 43 donors within 6 h postmortem. Notwithstanding technical challenges surrounding the RNA isolation from human retinas within a short timeframe, results from GSE160306 indicated that PTX3 gene expression was up-regulated in human retinas that exhibited both proliferative DR and DME, when compared to other experimental groups (Fig. 1*H*). Taken together, our findings demonstrated an increase in PTX3 in 9-mo mouse diabetic retinas and human diabetic retinas with complications. To identify the cell source(s) of PTX3 in the mouse diabetic retina, we explored the publicly available single-cell RNA-seq dataset GSE178121 that has been reported for the STZ-induced mouse diabetic model (20). PTX3 mRNA expression was found to be low or absent in most retinal cell types except for Muller cells (*SI Appendix*, Fig. S4 *A* and *B*). Muller cells in the STZ-induced mouse diabetic retinas exhibit PTX3 expression albeit at low levels and in a subpopulation of cells (*SI Appendix*, Fig. S4 *C* and *D*). We also harnessed the publicly available scRNA-seq dataset GSE135406 from the NMDA-induced retinal degeneration model, which damages inner retinal neurons (21), and combines acute neurodegeneration with severe sterile inflammation. As expected, rods, Muller glia, and bipolar cells were the retinal cells with higher frequencies (*SI Appendix*, Fig. S5*A*). Analysis of PTX3 mRNA expression revealed an acute time-response to NMDA in Muller glia, with the highest increase at 3 h after NMDA injection (*SI Appendix*, Fig. S5*B*). PTX3 mRNA levels decreased steadily after 3 h and went back to negligible expression similar to controls by 24 h (*SI Appendix*, Fig. S5 *C* and *D*).

### Decreased Reactive Gliosis in Diabetic Retinas of PTX3 Knockout Mice.

**Fig. 2.**
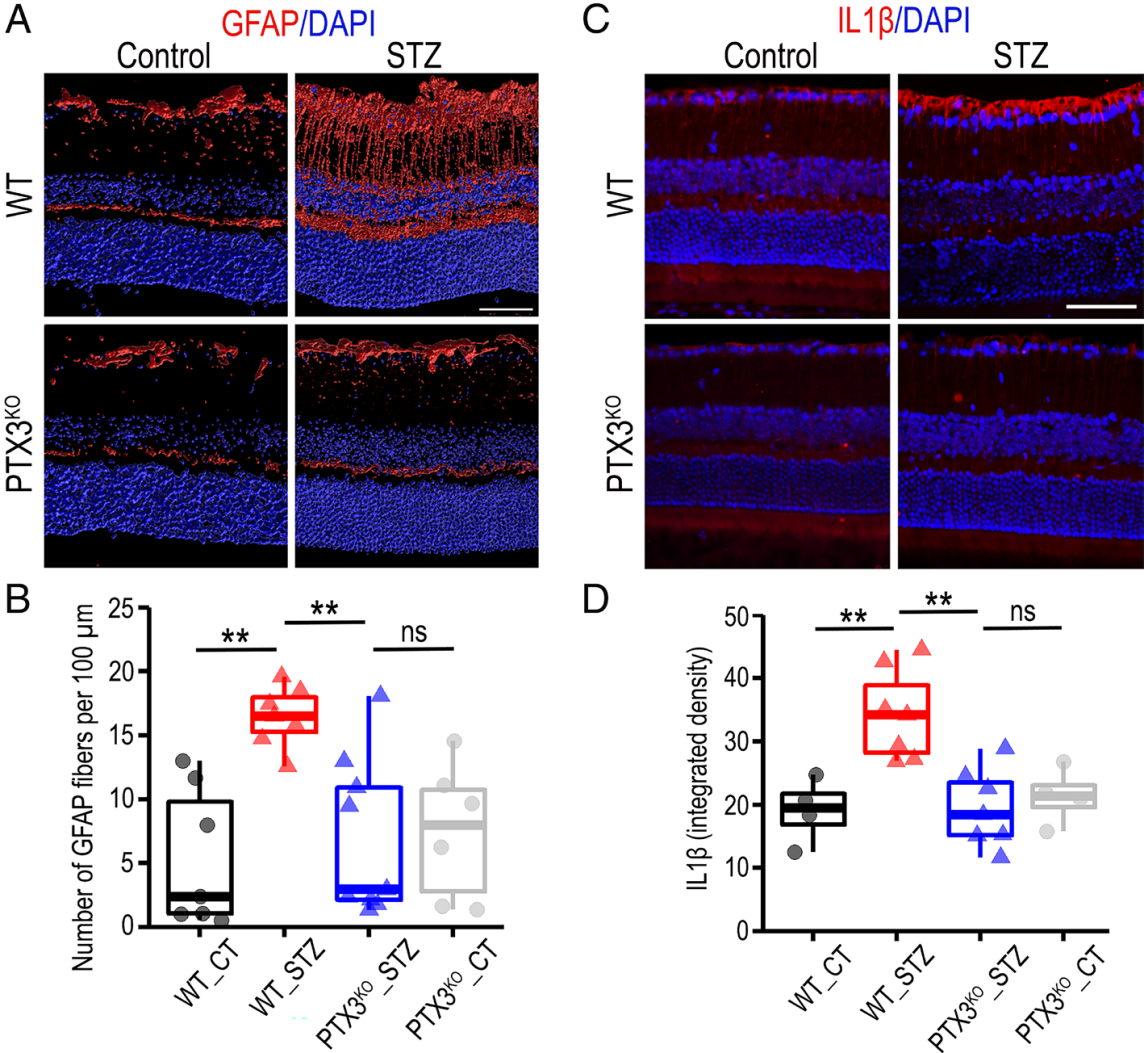
PTX3-deficient mice showed reduced reactive gliosis in diabetic retinas. (*A*) Immunofluorescence for GFAP (red) in retinal cross-sections from diabetic and nondiabetic mice, wild type, and PTX3^KO^. (Scale bar, 50 μm.) (*B*) Quantification of GFAP staining as number of fibers per 100 μm, **P < 0.01, n ≥ 6. (*C*) Immunostaining of mouse retinas with antibodies against IL1β (red). (Scale bar, 50 μm.) (*D*) Quantification of IL1β staining as integrated density, **P < 0.01, n ≥ 4.

To investigate the inflammatory hallmarks in diabetic retinas, we used the STZ-induced diabetes mouse model in PTX3 wild-type (WT) and knockout (PTX3^KO^) mice. After 9 mo of diabetes induction, immunohistochemistry of central retinal tissue showed that the number of GFAP fibers increased significantly in STZ diabetic retinas, when compared to nondiabetic animals (Fig. 2*A*). We found that PTX3^KO^ mice did not show such GFAP increase after 9-mo diabetes and were similar to nondiabetic animals (Fig. 2 *A* and *B*). Considering that the distribution of cell populations across the central and peripheral retina is different, we evaluated both areas of the retina. Results for GFAP staining in peripheral retinal areas were in agreement with those in central areas (*SI Appendix*, Fig. S6 *A* and *B*). We found a significant positive correlation between GFAP measurements in central and peripheral retinas (*SI Appendix*, Fig. S6*C*). In addition, we investigated IL1β expression and identified a significant increase in IL1β staining intensity in the retinal fiber layer with diabetes, which was not present in PTX3^KO^ mice (Fig. 2 *C* and *D*). These data provided evidence for a role of PTX3 in retinal reactive gliosis during diabetes.

### Retinal Reactive Gliosis Is Facilitated by PTX3.

**Fig. 3.**
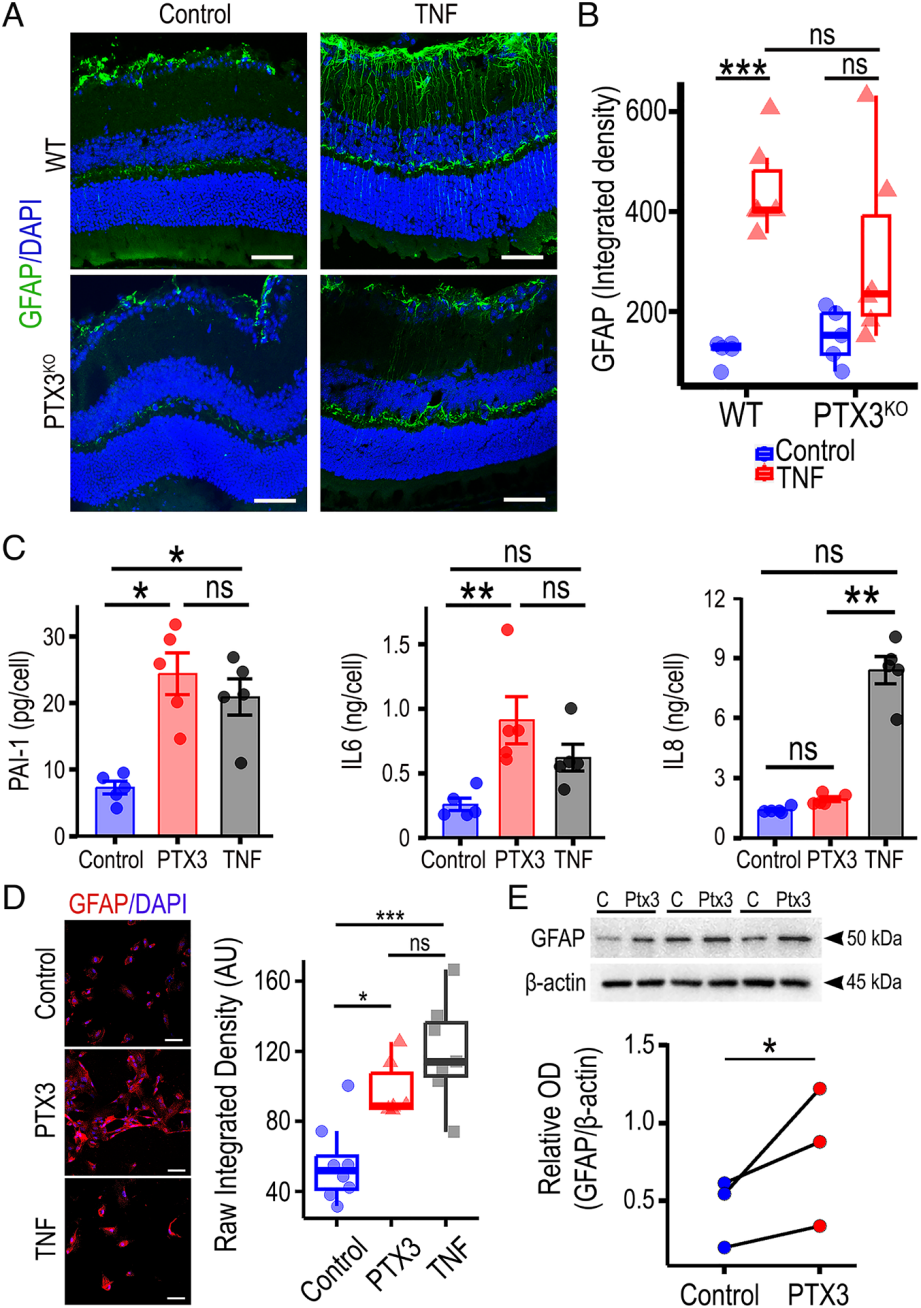
PTX3 induces activation of retinal macroglia. (*A*) Mouse retinal explants from WT or PTX3^KO^ mice were treated ex vivo with 5 ng/ml TNF-α for four hours and then stained for GFAP (red). (Scale bar, 50 μm.) (*B*) Quantification of GFAP signal in retinal explants using the raw integrated density per μm^2^ of retinal tissue. **P < 0.01; ns: nonsignificant, n ≥ 5. (*C*) Human retinal astrocytes were treated with vehicle, 5 ng/ml PTX3, or 5 ng/ml TNF-α, and the release of proteins PAI-1, IL6, and IL8 was quantified by ELISAs in culture supernatants collected after 72 h. *P < 0.05; **P < 0.01; ns: nonsignificant, n = 5. (*D*) Staining of retinal astrocyte cultures after treatments for GFAP (red). Quantification of GFAP staining was performed using the raw integrated density. *P < 0.05; ***P < 0.001; ns: nonsignificant, n ≥ 6. (Scale bar, 50 μm.) (*E*) Protein lysates from astrocyte cultures were assessed by western blotting, and ODs were used for relative quantification in relation to β-actin. *P < 0.05, n = 3.

To further confirm the role of PTX3 in enhancing reactive gliosis after an inflammatory stimulus, we treated retinal explants from PTX3^WT^ and PTX3^KO^ mouse ex vivo with recombinant TNF-α for 4 h. In line with previous results, there was a significant increase in GFAP staining in retinal explants from wild-type, but not in PTX3^KO^ retinas after TNF-α exposure (Fig. 3 *A* and *B*). The increased GFAP expression in the form of fibers spanning all retinal layers is characteristic of activated Muller glia. Furthermore, there was limited coexpression of CD31 with GFAP (*SI Appendix*, Fig. S7). This in addition to the spatial pattern of the GFAP distribution suggested Muller reactive gliosis. Gene expression changes were evaluated by RT-qPCR and we confirmed that PTX3 mRNA was significantly up-regulated after TNF-α treatment and, as expected, PTX3 mRNA was not found in PTX3^KO^ retinas (*SI Appendix*, Fig. S8). Similarly, Ccl2 mRNA was significantly up-regulated after TNF-α treatment in wild-type retinal explants, and this effect was abolished in PTX3^KO^ retinas. Interestingly, under control conditions, PTX3^KO^ retinas exhibited significantly lower expression of inflammatory genes Madcam1, Il1a, and Tlr2, when compared to wild-type retinas; however, under exposure to TNF-α, only Madcam1 was significantly reduced in the PTX3^KO^ retinas. In addition, we found no significant differences in the gene expression of Gfap and Ednrb across the experimental groups (*SI Appendix,* Fig. S8). Similarly, we also found that TNF-α significantly increased PTX3 expression in human retinal astrocytes (*SI Appendix,* Fig. S9). These results confirmed a role for PTX3 acting as a mediator in the inflammatory response in retinal macroglia. To identify the cell types that respond to PTX3, we screened human microglia, microvascular endothelial cells, and retinal astrocytes for IL6 release after PTX3 exposure. PTX3 treatment of retinal astrocytes induced a significant increase in IL6 release when compared to untreated controls, which was not observed in retinal endothelial cells and microglia (*SI Appendix,* Fig. S10). Investigation of PAI-1, IL6, and IL8 were chosen among various inflammatory proteins because cumulative evidence has shown these cytokine levels to be elevated in the diabetic retina (22, 23). Recently, they were found to be significantly up-regulated in retinas of diabetic patients presenting with active DME (24). Therefore, PAI-1, IL6, and IL8 have been suggested to be involved in the pathogenesis of DR (25). Focused evaluation of human retinal macroglia, with TNF-α treatment as a positive control, indicated that PTX3 significantly increased the secretion of PAI-1 and IL6, when compared to vehicle-treated controls (Fig. 3*C*), and such response was undistinguishable from TNF-α. On the other hand, IL8 secretion remained unchanged with PTX3 treatment but was significantly increased with TNF-α. Considering that the reactive gliosis in mouse diabetic retinas, characterized by GFAP upregulation, was abolished in PTX3^KO^ retinas both in vivo and ex vivo, we tested whether recombinant PTX3 promoted GFAP expression in human retinal astrocytes. Immunocytochemistry results confirmed a constitutive GFAP expression in human retinal astrocytes but also indicated that PTX3 boosted GFAP expression akin to TNF-α (Fig. 3*D*). Western blot corroborated that GFAP expression was significantly enhanced after PTX3 treatment (Fig. 3*E*). Similar results were found in mouse retinal mixed glia cultures obtained from the 16- to 17-wk-old db/db mouse. GFAP expression in diabetic mouse glia significantly increased after exposure to PTX3 (*SI Appendix,* Fig. S11 *A* and *B*). There was also a significant increase in IL6 release after PTX3 exposure which was comparable to the TNF-α-treated group (*SI Appendix,* Fig. S11*C*). These results recognized PTX3 as an important factor in mediating the inflammatory response in human and mouse retinal macroglia.

### PTX3 Deficiency Diminished Microglial Activation in Diabetic Retinas.

**Fig. 4.**
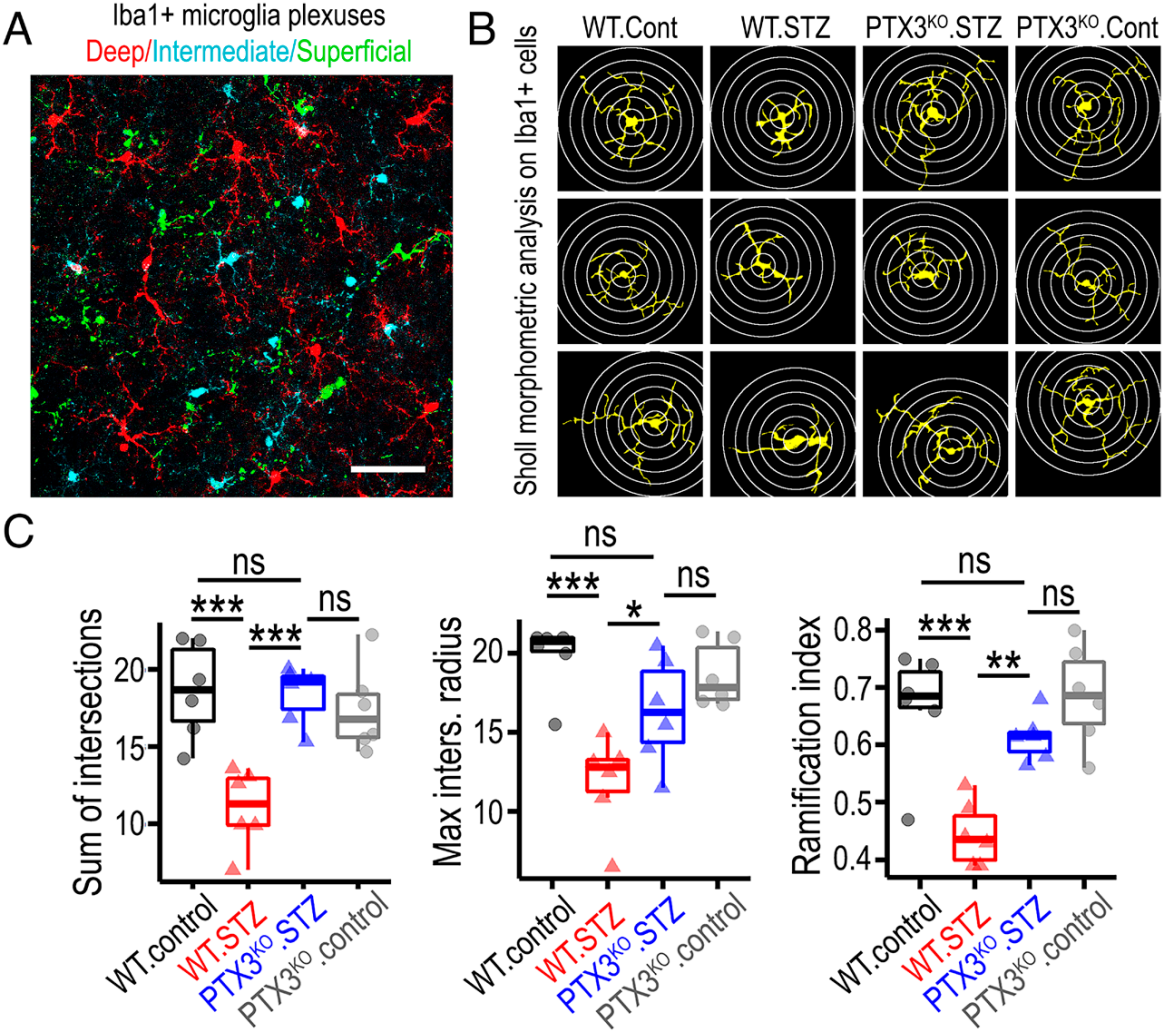
Retinal microglia activation is reduced in diabetic retinas lacking PTX3. (*A*) Microscopy image of retinal tissue stained for Iba1 and pseudocolored based on retinal depth obtained from z-scanned confocal microscopy to depict retinal layers as deep (red), intermediate (cyan), and superficial (green). (Scale bar, 50 μm.) (*B*) Representative images of microglia profiles as shown by Iba1 staining across the four experimental groups in the deep retinal layer. Concentric circles used in the Sholl morphometric analysis are shown in white. (*C*) Quantification of Sholl analysis metrics sum of intersections, maximal intersection radius, and ramification index in the deep retinal layer. *P < 0.05; **P < 0.01; ***P < 0.001; ns: not significant, n = 6.

Given the important role of microglia in the pathogenesis of DR, we next performed retinal microglial morphometric assessment using Iba1 immunostaining and the Sholl analysis. In order to characterize microglia and their phenotype at different anatomical locations, high-resolution fluorescent microscopy was performed across the retinal layers to identify microglia niches (Fig. 4*A*), as recently described (26). We found that the activated (ameboid) microglia phenotype, induced by diabetes, was most evident in the deep retinal layer (Fig. 4*B*), and these changes were less pronounced in the intermediate retinal layer and absent in the superficial retinal layer (*SI Appendix*, Fig. S12). As expected, a significant reduction in the sum of intersections, maximal intersection radius, and ramification index, were observed in diabetic WT when compared to nondiabetic WT retinas (Fig. 4 *B* and *C*). In the PTX3^KO^ mice, no differences were found between control and diabetic mice, and on the contrary, all the three Scholl parameters were significantly higher in the PTX3^KO^ diabetic retinas than in the WT diabetic retinas (Fig. 4*C*). To evaluate whether these microglia changes were associated with cell proliferation, we quantified microglia numbers, and found no significant differences across the experimental groups (*SI Appendix,* Fig. S13 *A* and *B*), suggesting that the diabetic environment induced a microglia phenotype change and not cell number expansion. These results revealed that the microglial activation induced by diabetes was inhibited in the PTX3^KO^ mice. Since PTX3 has been reported to inhibit phagocytosis of late apoptotic neutrophils by macrophages (27), we assessed whether recombinant PTX3 hinders the phagocytic activity of human microglia. It was found that PTX3 significantly inhibited phagocytosis of endothelial+astrocytic apoptotic bodies by human brain microglia (*SI Appendix,* Fig. S14 *A* and *B* and Movie S1). Taken together, these results are suggestive of a key role for PTX3 in retinal microglia activation during diabetes.

### Lack of PTX3 Protects Diabetic Retinas from Vascular Degeneration.

**Fig. 5.**
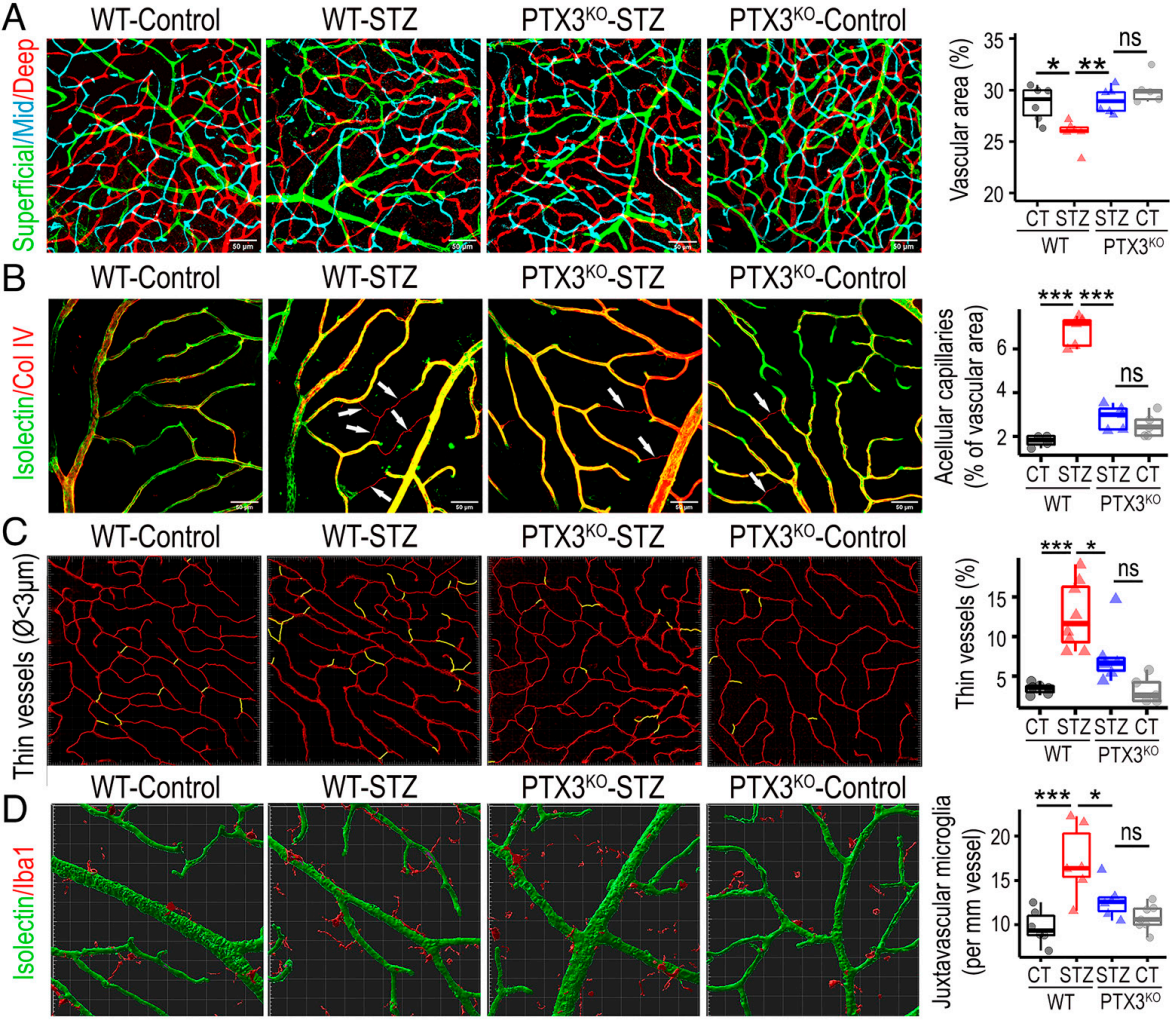
Diabetic mice lacking PTX3 were protected from retinal vasodegeneration. (*A*) Fluorescent confocal scanning microscopy image of retinal blood vessels stained with isolectin and pseudocolored based on retinal depth as deep (red), intermediate (cyan), and superficial (green). *P < 0.05; **P < 0.01; ns: not significant, n ≥ 5. (Scale bar, 50 μm.) (*B*) Immunostaining for Collagen IV (red) and isolectin (green) to identify and quantify acellular capillaries. Collagen IV–positive and isolectin-negative structures are indicated by white arrows. ***P < 0.001; ns: nonsignificant, n ≥ 4. (Scale bar, 50 μm.) (*C*) Imaris software-based analysis to select and quantify vasculature with a diameter smaller than 3 μm, defined as thin vessels. *P < 0.05; ***P < 0.001; ns: nonsignificant, n ≥ 5. (*D*) Costaining of retinas for Iba1 (red) and isolectin (green) in the superficial layer to identify and quantify juxtavascular Iba1+ cells. Each square block is 20 × 20 μm. *P < 0.05; ***P < 0.001; ns: nonsignificant, n ≥ 6.

To investigate a role for PTX3 in diabetic vascular pathogenesis, we evaluated a panel of vascular metrics including vascular density, acellular capillaries, nonperfused microvessels, and juxtavascular microglia. Isolectin staining and confocal microscopy were used to characterize the three vascular plexi in mouse retinas. We found that diabetic retinas exhibited significantly lower vascular densities than nondiabetic controls, and this phenotype was constrained in PTX3^KO^ animals (Fig. 5*A*). These changes were present across all retinal layers, but the vasodegeneration was the highest in the deep retinal plexus (*SI Appendix,* Fig. S15). We then evaluated the frequency of acellular capillaries, which were identified as Collagen IV–positive and isolectin-negative tubular structures. Results showed that the frequency of acellular capillaries significantly increased in diabetic retinas when compared to nondiabetic controls. On the contrary, acellular capillaries frequency in diabetic PTX3^KO^ retinas was not different from nondiabetics, while being significantly different from WT-diabetic retinas (Fig. 5*B*). High-resolution microscopy images of deep retinal plexus stained with isolectin were analyzed using Imaris software to single out microvasculature with a diameter smaller than 3 µm as a surrogate measure of nonperfused capillaries. We found that the frequency of thin microvessels of <3 µm, significantly increased in STZ diabetic retinas when compared to nondiabetic controls, and these changes were absent in PTX3^KO^ retinas (Fig. 5*C*). To assess the role of PTX3 in diabetes-induced vascular leakage, retinal tissues were stained and quantified for albumin. As expected, diabetes induced a significant increase in extravascular albumin (*SI Appendix,* Fig. S16*A*). The diabetic PTX3^KO^ retinas showed similar albumin staining levels to diabetic wild-type retinas (*SI Appendix,* Fig. S16*B*). Furthermore, we evaluated the transendothelial electrical resistance in human primary endothelial cells that were transfected with PTX3 shRNA lentivirus or control shRNA and treated with TNF-α to mimic an inflammatory milieu. Measurements were performed at frequencies of 1 and 41.5 kHz to enable the discrimination between cell barrier and cell coverage, respectively. Results from readings at 1 kHz highlighted that TNF-α significantly diminished barrier function (*SI Appendix*, Fig. S16*C*). Interestingly, PTX3 knockdown cells exhibited consistently higher impedance than control and TNF-α-treated PTX3 knockdown cells at 1 kHz. These results were not seen at 41.5 kHz. In addition, after exposure to Thrombin, we found no significant difference between control and PTX3 knockdown cells treated with TNF-α; but impedance impairment was significantly lower in PTX3 knockdown cells (*SI Appendix*, Fig. S16*D*). In summary, these results indicated that PTX3 does not play a major role in the blood–retinal barrier breakdown in vivo model, however, lack of PTX3 was associated with higher transendothelial electrical resistance at 1 kHz in the in vitro model. In addition, the frequency of juxtavascular Iba1+ cells was evaluated in the retinal superficial vascular plexus. Iba1-positive cells located next to isolectin-labeled vasculature, increased significantly in STZ diabetic mice when compared to controls, and this diabetes-related increase in juxtavascular Iba1+ cells was not found in PTX3^KO^ retinas (Fig. 5*D*). Moreover, 3D surface–surface colocalization analysis showed that the surface area of contact between isolectin and Iba1-positive 3D structures was higher in diabetic wild-type than in diabetic PTX3^KO^ retinas (*SI Appendix,* Fig. S17). Taken together, these results indicated that PTX3^KO^ mice were protected from diabetes-induced vasodegeneration.

### Visual Function Is Preserved in Diabetic Mice Lacking PTX3.

**Fig. 6.**
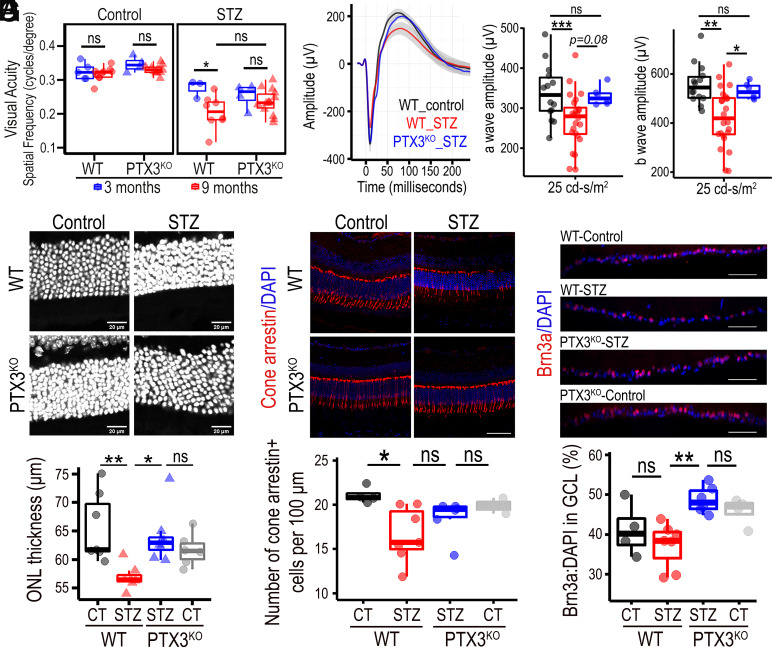
Retinal function is protected in diabetic retinas lacking PTX3. (*A*) Optokinetic responses measured at 3 and 9 mo since diabetes induction in WT and PTX3^KO^ mice. Age-matched nondiabetic animals were used as controls. *P < 0.05; ns: not significant, n ≥ 5. (*B*) Electroretinograms performed at 9-mo diabetes. Trace shows the mean for the experimental group with CI in gray. (*C*) Quantification and statistical analysis of a wave from electroretinograms. ***P < 0.001; ns: not significant, n ≥ 4. (*D*) Statistical comparison of the b wave from electroretinograms. *P < 0.05; **P < 0.01; ns: not significant, n ≥ 4. (*E*) Imaging and evaluation of the outer nuclear layer in retinas stained with DAPI for nuclear identification. *P < 0.05; **P < 0.01; ns: not significant, n ≥ 6. (Scale bar, 20 μm.) (*F*) Immunostaining for Cone arrestin (red) with quantification of the number of cells per 100 μm. *P < 0.05, ns: not significant, n ≥ 2. (Scale bar, 50 μm.) (*G*) Staining of retinas for Brn3a (red) and quantification as a percentage of Brn3a-positive cells in total cells identified by DAPI nuclear staining. **P < 0.01; ns: not significant, n ≥ 4. (Scale bar, 50 μm.)

Having established that the PTX3^KO^ retinas did not exhibit the prototypical diabetes-induced histopathological changes in macroglia, microglia, and vasculature, we next investigated whether such differences could protect retinal physiology and thereby preserve visual function. We evaluated optokinetic responses, which confirmed a significant decrease in visual acuity between 3 and 9 mo in diabetic animals; on the contrary, nondiabetic animals did not show such a decrease in visual acuity. Interestingly, PTX3^KO^ mice did not exhibit the diabetes-induced decrease in visual acuity (Fig. 6*A*). In agreement with these results, scotopic electroretinograms revealed a significant decrease in the amplitudes of a and b waves in STZ diabetic animals when compared to nondiabetic controls (Fig. 6*B*). In diabetic mice, the amplitude of the a wave was not different between wild-type and PTX3^KO^ retinas (Fig. 6*C*); but the amplitude of the b wave in PTX3^KO^ diabetic retinas was higher than in wild-type diabetic retinas, and similar to the amplitudes found in nondiabetic mice (Fig. 6*D*). To further investigate diabetes-induced neurodegeneration, we evaluated the thickness of the outer nuclear layer (ONL) where photoreceptor nuclei are located. This confirmed a significant decrease in ONL thickness in diabetic retinas when compared to nondiabetic controls, which was not found in PTX3^KO^ retinas (Fig. 6*E*). We then performed immunohistochemistry for cone-arrestin, Brn3a, and synaptophysin to investigate cones, retinal ganglion cells, and retinal neuronal cells, respectively. We found that the significant decrease in cone-arrestin-positive cells observed during diabetes in wild-type animals, was not observed in PTX3^KO^ animals; however, there was no difference between diabetic wild-type and diabetic PTX3^KO^ retinas (Fig. 6*F*). In addition, the frequency of Brn3a^+^ retinal ganglion cells in diabetic retinas was significantly higher in PTX3^KO^ than in wild-type retinas (Fig. 6*G*). Interestingly, we found no differences across experimental groups when synaptophysin was evaluated (*SI Appendix,* Fig. S18), and there were no significant changes in total retinal thickness from OCT images (*SI Appendix,* Fig. S19) or cross-retinal sections stained for H&E (*SI Appendix,* Fig. S20), between the wild-type and PTX3^KO^ diabetic mice. We also evaluated the impact of the PTX3^KO^ genotype in glucose metabolism by measuring HbA1c and glycemia levels at 9 mo after diabetes induction. The nondiabetic PTX3^KO^ and wild-type mouse had similar HbA1c and glycemia. While there was no significant difference in HbA1c between the diabetic PTX3^KO^ and wild-type mouse, there was a significant small fold-change decrease in glycemia levels. Results showed that diabetes induction caused a mean difference of 21 mmol increase in glycemia, while the PTX3^KO^ genotype was associated with a mean difference of 4 mmol decrease in diabetic mice (*SI Appendix,* Fig. S21). In summary, PTX3 deficiency during diabetes contributed to preservation of visual function and delay of neurodegeneration, without affecting HbA1c levels.

## Discussion

PTX3 plays a key role in limiting inflammation in various hematopoietic cell models (28, 29). However, the PTX3 actions can be nuanced and it has been suggested to promote inflammation in models of obesity (30), rheumatoid arthritis (31), and renal ischemia/reperfusion injury (32). We show evidence to demonstrate that PTX3 acts as a molecular driver of DR as demonstrated by PTX3-deficient mice being protected from developing key facets of DR, including reactive gliosis, microglia activation, and vascular and neural degeneration. Importantly, all these translated into preserved visual function in the PTX3^KO^ mouse. We attributed this to the reduced retinal sterile inflammation and consequent diminished vaso-neurodegeneration in PTX3^KO^ mice, which protected optokinetic and electroretinogram (ERG) responses from impairment under diabetic conditions. Interestingly, our analysis highlighted that the largest vasodegeneration and microglia activation were found in the deep retinal layers. This is congruent with the lowest oxygen saturation levels found at the deep retinal layers (33).

PTX3 is a pattern recognition molecule produced by different cell types such as fibroblasts, endothelial cells, macrophages, and glia (34). In the mouse retina, we found PTX3 protein accumulation in the nerve fiber, outer nuclear, and outer plexiform layers, but only at 9-mo diabetes. This agrees with a significant increase in PTX3 gene expression in the human retina, but only in the presence of both late-stage DR complications NPDR and DME. The PTX3 protein staining profile suggests proximity to Muller cells, which is in line with publicly available scRNA-seq data, indicating Muller cells as the major source of PTX3 transcript in the mouse retina. Based on mouse scRNA-seq data and in vitro human retinal cell cultures, we pinpointed the retinal macroglia as an important cell type that transcribes and responds to PTX3. The expression of PTX3 is triggered by infection, tissue damage, and dysmetabolism as part of the innate immune response (7). At the mRNA level, and based on scRNA-seq data, we confirmed the PTX3 local upregulation as an early response to tissue damage in the murine retina following exposure to NMDA. In this model of excitotoxicity-induced retinal injury, PTX3 gene expression in Muller glia was significantly up-regulated within 3 h following NMDA exposure, which was then followed by reversion to negligible expression by 72 h after injury (*SI Appendix,* Fig. S5). Diabetes-induced changes to the retinal microenvironment are known to be highly nuanced depending on the diabetes type, diet, glucose excursions, light exposure, and circadian rhythm. From our data, it is reasonable to suggest that the diabetic retina experiences a higher frequency of bouts of PTX3 mRNA expression in response to localized neuroinflammatory events when compared to the nondiabetic retina. PTX3, as an acute phase protein, is released during insult and accumulates within the retinal neuropile, as we describe in our diabetic mouse model at 9 mo after STZ injection. Such tissue accumulation of PTX3 in inflamed tissues is facilitated by its capacity to incorporate into extracellular matrices (18). Thus, our findings propose different kinetics for PTX3 expression in the diabetic retina. While gene expression is acute, local, and transitory in cells exposed to injury; the PTX3 protein accumulates extracellularly and may exacerbate tissue damage over prolonged time frames.

While extensive evidence has demonstrated that PTX3 is expressed in several leukocytes, including monocytes, macrophages, dendritic cells, and neutrophils, in the context of infection and sepsis (35, 36), studies in circulating blood for PTX3 levels in diabetes are inconclusive (13). Nevertheless, PTX3 was elevated in the aqueous humor of patients with diabetes when compared to the nondiabetic cohort (17). Interestingly, among diabetic patients, there was no difference between patients with and without DR. These findings support a role for PTX3 in the early stages of DR, which reflects on the increased PTX3 levels found in the local eye microenvironment. This aligns with our results, and thus, we propose that PTX3 is important for the development of the complications of DR. Moreover, IL1β and TNFα which contribute to the diabetic inflammatory microenvironment, are known to effectively promote PTX3 expression in macrophages, endothelial cells, and retinal pigmented epithelium (13). In light of all this evidence, we suggest that PTX3 functions at the crossroads of inflammation and diabetes.

Our data also provide mechanistic insight into the role of PTX3 in the proinflammatory retinal microenvironment as diabetes progresses. PTX3 alone was sufficient to induce a gliosis-like response in human retinal macroglia in vitro, characterized by GFAP upregulation alongside secretion of PAI1 and IL6. We also showed that PTX3 inhibited phagocytosis in microglia, which may lead to accumulation of cell debris that contributes to enhancing sterile inflammation. Inhibition of phagocytosis in myeloid cells by PTX3 has been shown in other models (27). In this regard, PTX3 has been reported to sequester C1q (37), a major recognition unit of the classical complement pathway, that binds to apoptotic cells promoting their phagocytosis. C1q is also present in the DR eye (38). Similarly, as evidence demonstrates that RPE express PTX3 (13), it is possible that PTX3 also controls RPE phagocytosis of photoreceptor outer segments, which provides another therapeutic target in DR and AMD pathogenesis. Microgliosis in DR has recently been linked to fibrinogen deposition (39), and PTX3 has the capacity to bind fibrinogen, orchestrating tissue remodeling (40). In addition, we showed an increase in juxtavascular microglia with diabetes that was diminished in the PTX3^KO^ mouse. All these findings suggest a potential link between PTX3, fibrinogen, and microglia activation. Moreover, tenascin-R, aggrecan, and hyaluronic acid are involved in the assembly of the extracellular matrix of perineuronal nets (41), PTX3 has been reported to incorporate into hyaluronan-rich extracellular matrix (18), and interestingly we found PTX3 accumulation in the outer plexiform layer of diabetic retinas. Also, the extracellular protein Tenascin-R has been reported to increase in the OPL in a mouse model of retinal ischemia (42). It is therefore conceivable that PTX3 accumulation in the diabetic retina is, to some extent, dependent on its interaction with extracellular matrix proteins. Such tissue accumulation of PTX3 protein expression in inflamed tissues is facilitated by its capacity to incorporate into extracellular matrices and this is in agreement with our findings showing close association of PTX3 with Tenascin-C and Endostatin in the OPL; and with Versican and biotinylated hyaluronic acid binding protein in the ILM. The binding of PTX3 to ECM should be highlighted as this suggests that PTX3 protein extraction from tissues like the retina may require nonstandard protocols, as previously described (18).

PTX3 was shown to inhibit angiogenesis by binding to FGF2 (43, 44), and our results confirmed that PTX3 deficiency protected mouse retinal vasculature from degeneration induced by a diabetic milieu. Our results showed PTX3 involvement in various aspects of DR although this was not evident in compromise of the inner blood–retinal barrier. Vascular leakage was increased in diabetic retinas although deletion of PTX3 did not alter this response. It is important to note that while the STZ mouse model represents early-stage DR and there is no retinal edema leading to vision loss. In addition, mice do not have a macula, and therefore the direct relationship between DME and vision impairment is not possible in mouse models. On the other hand, the gliosis response in 9-mo STZ mouse retinas was unequivocal and consistent. Our data showed convincingly that modulation of inflammation in Muller macroglia by PTX3 is a strong driver of visual impairment in the mouse STZ diabetic model. Moreover, the microglia activation and IL-1β increase, reported in ischemic rat retinas (45), was confirmed in the mouse 9-mo diabetic WT, but not in the PTX3^KO^ retinas. In the context of DR, PTX3 has been suggested as an inflammatory biomarker, but there is conflicting evidence (13). This lack of consistency is likely due to the complex dynamics of PTX3 transcription, translation, and secretion. PTX3 is an acute phase protein, reported to be produced by local tissue-resident cells under stress and accumulate within inflamed tissues. In the mouse retina, we have shown that PTX3 mRNA transcription is acutely activated in Muller cells after retinal injury. Our results also demonstrated that PTX3, a member of the acute phase proteins, accumulates in the diabetic retina and enhances sterile inflammation. These findings have important implications for advanced therapies targeting inflammatory processes in DR.

## Materials and Methods

### Animals.

The PTX3-knockout mice (PTX3^KO^) on C57BL/6 J background were provided by Cecilia Garlanda, IRCCS Humanitas Research Hospital. Generation of PTX3^KO^ mice was previously described (46). All animal experiments were conducted in accordance with the UK Home Office regulations and the Association for Research in Vision and Ophthalmology. Diabetes was induced in male mice at the age of 12 to 14 wk using five daily i.p. injections of streptozotocin (Merck Life Sciences UK Ltd.) at a concentration of 50 mg/kg body weight.

### Immunohistochemistry.

Retinas were fixed in 4% paraformaldehyde. Whole-mounted retinas were incubated with isolectin B4-biotin conjugate (20 μg/ml, Sigma-Aldrich UK; catalog L2140) and recombinant anti-Iba1 antibody followed by labeling with streptavidin AlexaFluor 488 or AlexaFluor 594 (1:500; Invitrogen, UK). A subset of fixed retinas was embedded in OCT or paraffin and sectioned at 10 µm thickness. Details of the primary antibodies used and their dilutions are listed in *SI Appendix*, Table S1. Image acquisition was performed using Leica SP5, SP8, and Leica Stellaris-5 confocal microscopes. Image analysis was carried out using Fiji-Image J2, Leica LasX, or Imaris (Oxford Instruments, UK).

### Ex vivo assay with retinal explants.

Eyes were enucleated from mice and transferred to a petri dish containing DMEM + 5% FBS. Two lateral incisions were made intravitreally using 29G needles, and eyes were incubated with 5 ng/ml human recombinant TNFα (Novus biologicals, UK) at 37 °C and 5% CO_2_. After 4-hour incubation, the eyes were fixed in 4% paraformaldehyde and embedded in OCT for immunostaining for GFAP. Image analysis was performed using ImageJ and raw integrated density, defined as the product of area and fluorescence intensity, which was normalized per μm^2^ of retinal tissue, considered between the nerve fiber layer and the photoreceptor outer segments.

### Cells.

Primary human retinal astrocytes (Innoprot, Spain) were grown on Poly-L-Lysine (Sigma-Aldrich UK, P4707) coated flasks and cultured in astrocyte medium (Innoprot, Spain), supplemented with 10% FBS. Primary human retina microvascular endothelial cells (Innoprot, Spain) were cultured on fibronectin from human plasma (Sigma-Aldrich, UK) coated flasks using endothelial cell medium (ECM, Innoprot, Spain), supplemented with 5% FBS. Human brain microglia cells (HMC3) purchased from ATCC (CRL-3304) were grown on uncoated flasks in EMEM (Thermo Fisher Scientific) supplemented with 10% FBS.

### Immunocytochemistry.

Cells grown on coverslips were fixed with 4% PFA for 15 min at room temperature or 100% ice-cold methanol for 15 min at -20 °C. Fixed cells were stained with primary antibody for GFAP (MA5-12023, ThermoFisher Scientific, UK) or PTX3 (affinity purified rabbit polyclonal IgG anti-human PTX3 developed in-house) overnight at 4 °C in a humidified chamber. Coverslips were washed three times for 5 min each with PBS 0.1% Triton-X (PBST) and incubated with goat anti-mouse AlexaFluor 594 or donkey anti-rabbit AlexaFluor 594 (1:500; Invitrogen, UK) at 37 °C for 1 h in a humidified chamber. After three washes for 5 min in PBST, coverslips were mounted on glass slides with Vectashield with DAPI for nuclear staining. Stellaris 5 microscope was used for imaging. Fiji-Image J2 software was used for analysis.

### Imaging and quantification.

Confocal Z-stacks were generated by scanning through the whole retinal depth (60 to130 µm). Morphological analysis of Iba1-positive cells was performed using Leica LASX, Fiji-Image J2, and Sholl analysis plugin. Iba1-positive cells were categorized based on their overall depth location as superficial, intermediate, and deep. For this, we also used three distinct isolectin B4-positive vascular layers as location references. Iba1+ cells were processed for Sholl analysis. A single point was placed at the center of the cell soma; the starting and ending radii were set as 10 and 100 µm, respectively, with a 10 µm step size. Sholl analysis readouts include sum intersections, mean intersections, maximum intersections, maximum intersection radii, ramification index, and centroid radii. For each retinal wholemount, we measured up to twenty Iba1+ cells per central or peripheral retinal area. Maximum intensity projections were prepared for isolectin B4^+^ retinal vasculature corresponding to superficial, intermediate, and deep vasculature plexuses. Individually separated vascular plexuses were binarized and used to measure vascular density and branching complexity using Angiotool software.

### Optokinetics.

Optokinetic assessments were performed in 3- and 9-mo diabetic mice and age-matched control animals. WT and PTX3^KO^ mice were assessed. The day before testing, animals were placed for at least one hour into the room with the Optometry box, for adaptation to the environment. Mice were placed on the testing platform inside the Optometry box. Optokinetic responses were measured by tracking head movements during the projection of a virtual rotating drum.

### Electroretinogram.

Full-field scotopic Ganzfeld electroretinography (ERG) was performed using an Espion visual electrophysiology system (Diagnosys LLC, Littleton, MA), according to the manufacturer’s instructions. Briefly, mice were dark-adapted overnight, and the procedures were conducted under dim-red light (<1 lx). Prior to anesthesia via intraperitoneal injection with Ketamine and Rompun, mouse pupils were dilated with 2.5% phenylephrine hydrochloride and 1% atropine sulfate (Chauvin, Essex, UK) and moisturized with Viscotears Liquid Gel (Novartis Pharmaceuticals Ltd., Surrey, UK). Corneal ERG electrodes recorded four responses that were averaged at each light intensity (0.008, 0.025, 0.08, 0.25, 0.8, 2.5, 8, and 25 cdxs/m^2^). A-wave and B-wave amplitudes were measured using Espion analysis software (Diagnosys Technologies, Littleton, MA).

### Transcriptomics from Public Databases.

For the RNAseq dataset GSE160306 (19), raw table of counts were retrieved from GEO and reanalyzed in R using DESeq2 standard workflow. Specific comparisons were selected, and DEGs were defined setting a cutoff of FDR ≤ 0.05 and absolute log2 FC > 1. Overall results obtained from the reanalysis are in keeping with one presented in the original paper. We then focused our attention on the estimates of PTX3 expression. Single-cell RNAseq data from NMDA treated and control mice, STZ-treated and controls, were retrieved from a published studies with raw counts available at GSE135406 (21) and GSE178121 (20), respectively. Seurat objects of the filtered cells’ barcodes were downloaded from the original paper. Briefly, we relied on the whitelist of the cell barcodes provided by the authors to filter only the cell-associated barcodes. The counts were processed with the standard workflow of Seurat (4.1.1) for sample integration. We have integrated ten individual samples (2 controls and 8 NMDA treated) for a total of 38,305 cells. Our reanalysis overlaps well with the results shown in the original paper. We decided to apply the cell annotation provided by the authors. The final integrated object has been used for further data analysis, exploring the expression of PTX3 across treatments and times.

### ELISAs.

Cells were cultured in 24-well plates on glass coverslips. Cell culture supernatant was collected after the treatment of human retinal astrocytes, HMC3 microglia, and HRMECs with 5 ng/ml recombinant human PTX3 (Enzo Lifesciences, UK) and human recombinant TNFα (Novus biologicals, UK) for 72 h. Extracellular release of IL6, IL8, and PAI-1 was quantified in 96-well plates using ELISA kits (R&D Systems, UK). The absorbance was read using a PHERAstar microplate reader, and concentrations for each molecule were calculated using the four-parameter logistic equation, based on standard curves performed following manufacturer instructions. Cells on coverslips were then fixed with 100% ice-cold methanol for 15 min at −20 °C and mounted on glass slides using Vectashield with DAPI (Vector laboratories) for normalization to cell density.

### Western Blot.

Human retinal astrocytes were treated with 5 ng/ml recombinant human PTX3 (Enzo Lifesciences, UK) or 5 ng/ml human recombinant TNFα (Novus biologicals, UK), or vehicle control, for 72 h. After treatment, cells were washed once with PBS, and protein was extracted as follows. RIPA buffer (1X) with EDTA, protease, and phosphatase inhibitors (ThermoFisher Scientific) were used to lyse cells. Protein concentrations were obtained using the Pierce BCA Protein Assay Kit (ThermoFisher Scientific). Electrophoresis was performed using 20 µg of protein, which was then transferred to PVDF membranes. Blocking was performed for 1 h at room temperature using 5% nonfat dry milk (Santa Cruz Biotechnology) in TBS with 0.1% Tween-20 (vol/vol, TBST), followed by overnight incubation of primary antibodies at 4 °C. Primary antibodies were prepared using 1X Clear Milk (ThermoFisher Scientific). Antibodies against GFAP (ThermoFisher Scientific MA5-12023) were used at 1:500, and against β-actin (Cell Signaling 5125) at 1:1,000. After washing with TBST, membranes were incubated for 1 h at room temperature with horseradish peroxidase–conjugated (HRP) secondary antibodies (Bio-Rad), made in 1X Clear Milk at 1:3,000 dilution. After washing in TBST, chemiluminescence HRP substrate (Bio-Rad) and the G:BOX instrument (Syngene) were used to develop and image the blots, respectively. The immunoblots were then quantified and analyzed using Fiji-Image J2 software.

### Phagocytosis Assay.

Human microglia cells were seeded at a density of 5,000 cells per well in a clear bottom 96-well plate. Human endothelial cells (5 × 10^5^) and human retinal astrocytes (5 × 10^5^) were treated with 1 μM staurosporine to induce apoptosis. After 16 h incubation with staurosporine, apoptotic bodies were collected in a sterile tube using centrifugation for 10 min and then resuspended in 1 ml of DMEM + 10% FBS. Apoptotic bodies were incubated with pHrodo™ Red, succinimidyl ester (pHrodo™ Red, SE, Thermo Fisher Scientific) for 1 h at 37 °C + 5% CO_2_. After two washes with PBS, the pHrodoSE-tagged apoptotic bodies were resuspended in microglia media prior to their addition on top of microglia culture in a 96-well plate. For nuclear visualization, a single drop of NucBlue™ Live ReadyProbes™ (Thermo Fisher Scientific) reagent was added to each 96-well. Image acquisition was performed using a Leica TIRF microscope, and analysis was carried out using LASX software.

### Statistical Analysis.

Biological replicates were used in all experiments with sample size shown in figure legends. Data are represented as mean ± SEM, and statistical significance for comparison between two groups was evaluated using Prism software and unpaired two-tailed *t* test analysis. For multiple comparisons, we used one-way ANOVA testing. A value of *P < 0.05, **P < 0.01, and ***P < 0.001 was considered significant.

## Supplementary Material

Appendix 01 (PDF)

Movie S1. **Recombinant PTX3 diminishes phagocytosis activity in human microglia**. Time-lapse live cell imaging of human microglia HMC3 cocultured with pHrodo succinimidyl ester red apoptotic bodies. Apoptotic bodies were generated from human retinal microvascular endothelial cells and human retinal astrocytes treated with Staurosporine. Images were taken using a TIRF microscope every 12 minutes up to 4 hours. Red signal indicates intracellular accumulation of apoptotic bodies within microglia cells.

## Data Availability

All study data are included in the article and/or supporting information. Previously published data were used for this work [GSE160306: (19). GSE178121: (20, 21)].
