# Supplementary material for: Modulation of diabetes-related retinal pathophysiology by PTX3
Source: Proc Natl Acad Sci U S A. 2024 Sep 30;121(41):e2320034121. doi: 10.1073/pnas.2320034121 (PMC11474045; doi:10.1073/pnas.2320034121)
Supplement: Supplementary file 1 — Appendix 01 (PDF) [file pnas.2320034121.sapp.pdf]

PNAS

Supporting Information for
Modulation of diabetes-related retinal pathophysiology by PTX3

Varun Pathak, Pietro M. Bertelli, Edoardo Pedrini, Kevin Harkin, Elisa Peixoto, Lynsey-Dawn Allen, Kiran Mcloughlin, Natasha D. Chavda, Kevin J. Hamill, Jasenka Guduric-Fuchs, Antonio Inforzato, Barbara Bottazzi, Alan W. Stitt, Reinhold J. Medina.

Reinhold J. Medina
Email: r.medina-benavente@liverpool.ac.uk

This PDF file includes:

Figures S1 to S21
Table S1
Legend for Movie S1

Other supporting materials for this manuscript include the following:

Movies S1

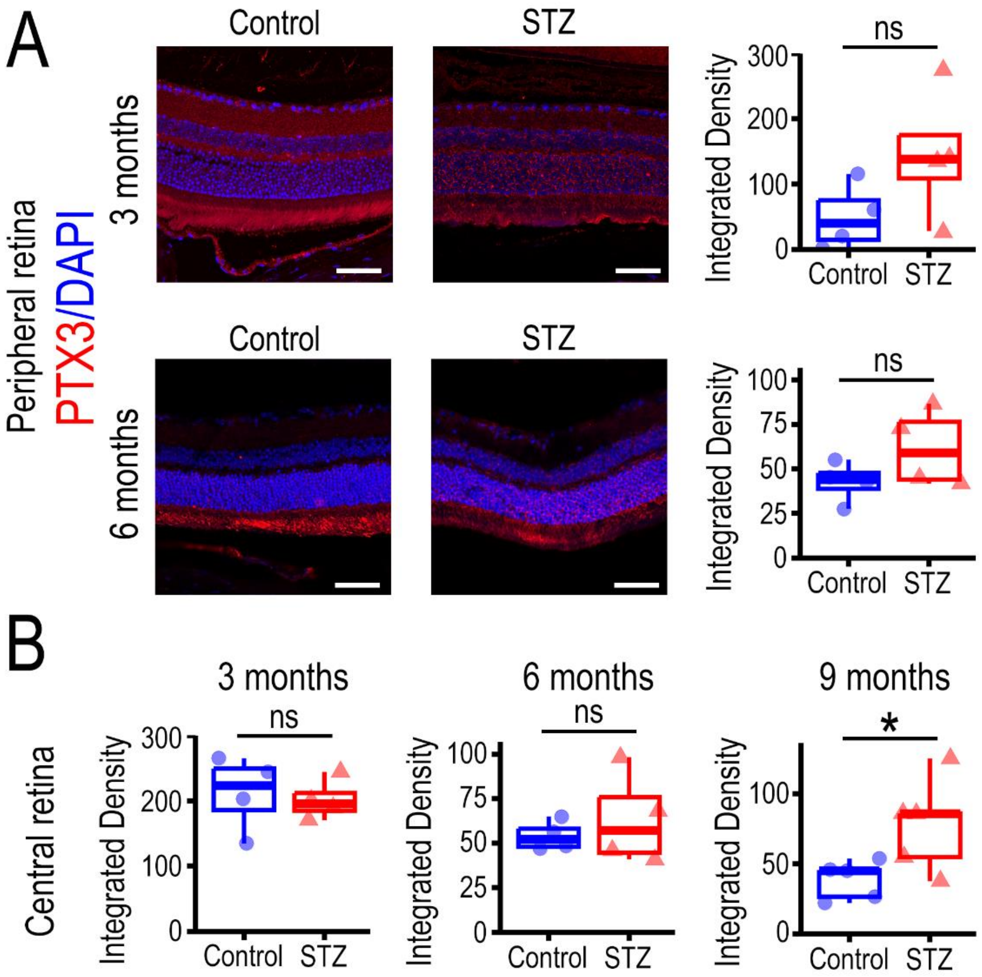

Fig. S1. PTX3 expression at different timepoints in the retina of diabetic mice and non-diabetic littermates. (A) Immunohistochemistry for PTX3 (red) in retinal cross sections of peripheral mouse retinas at 3- and 6-month post diabetes induction with STZ compared to age-matched controls. Nuclei are stained with DAPI (blue). Area of positive staining for PTX3 was quantified as integrated density per μm^2 of retinal tissue, n = 4, ns: not significant. Scale bar: 50 μm. (B) Quantification and statistical analysis of PTX3 immunostaining in central mouse retinas at 3, 6, and 9 months post-STZ injection, n≥4, ns: not significant, *p<0.05.

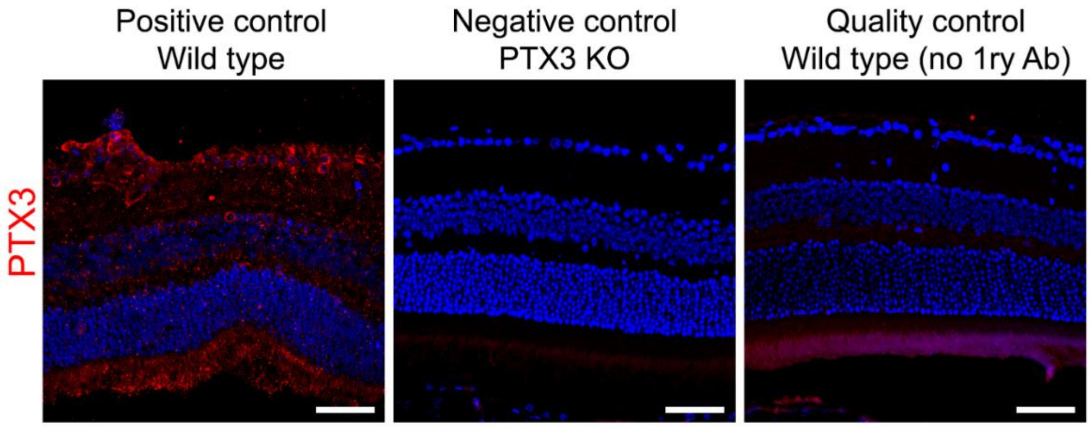

Fig. S2. PTX3 staining in retinal tissue from PTX3 knockout mouse as negative control. A retinal tissue cross section from PTX3 knockout mouse was used as a negative control. As the quality control for background signal, a wild-type retina was stained with 2ry antibody only without the 1ry antibody against PTX3. Scale bar: 50 μm.

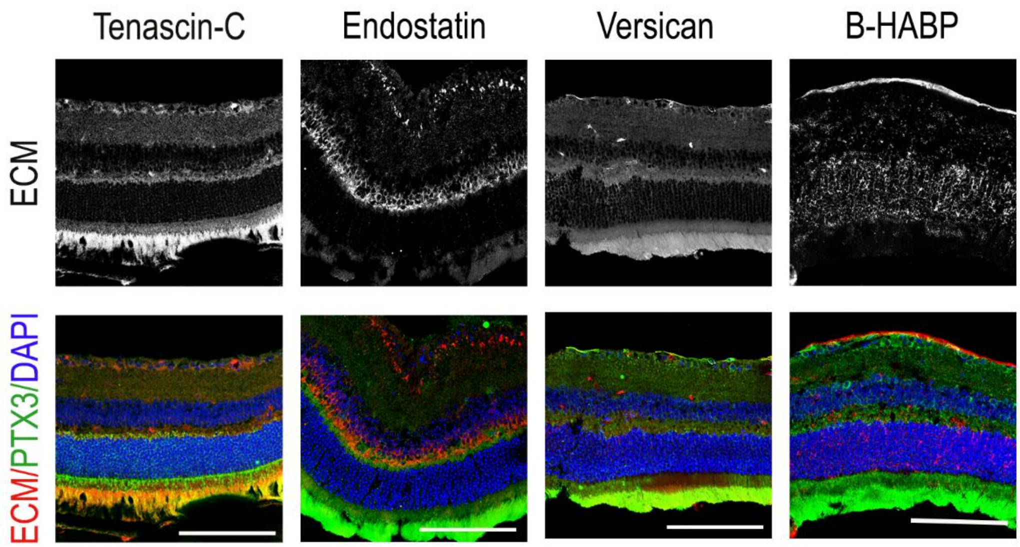

Fig. S3. Immunostaining for extracellular matrix (ECM) proteins and PTX3 in mouse diabetic retinal tissue. Retinal tissue cross sections from 9-month diabetic mice were evaluated for the expression of PTX3 and ECM proteins Tenascin-C, Endostatin, Versican, and biotinylated hyaluronic acid binding protein (B-HABP). Upper panels show ECM staining in white, and lower panels the merged image with ECM (red), PTX3 (green) and DAPI (blue). Scale bar: 100 µm.

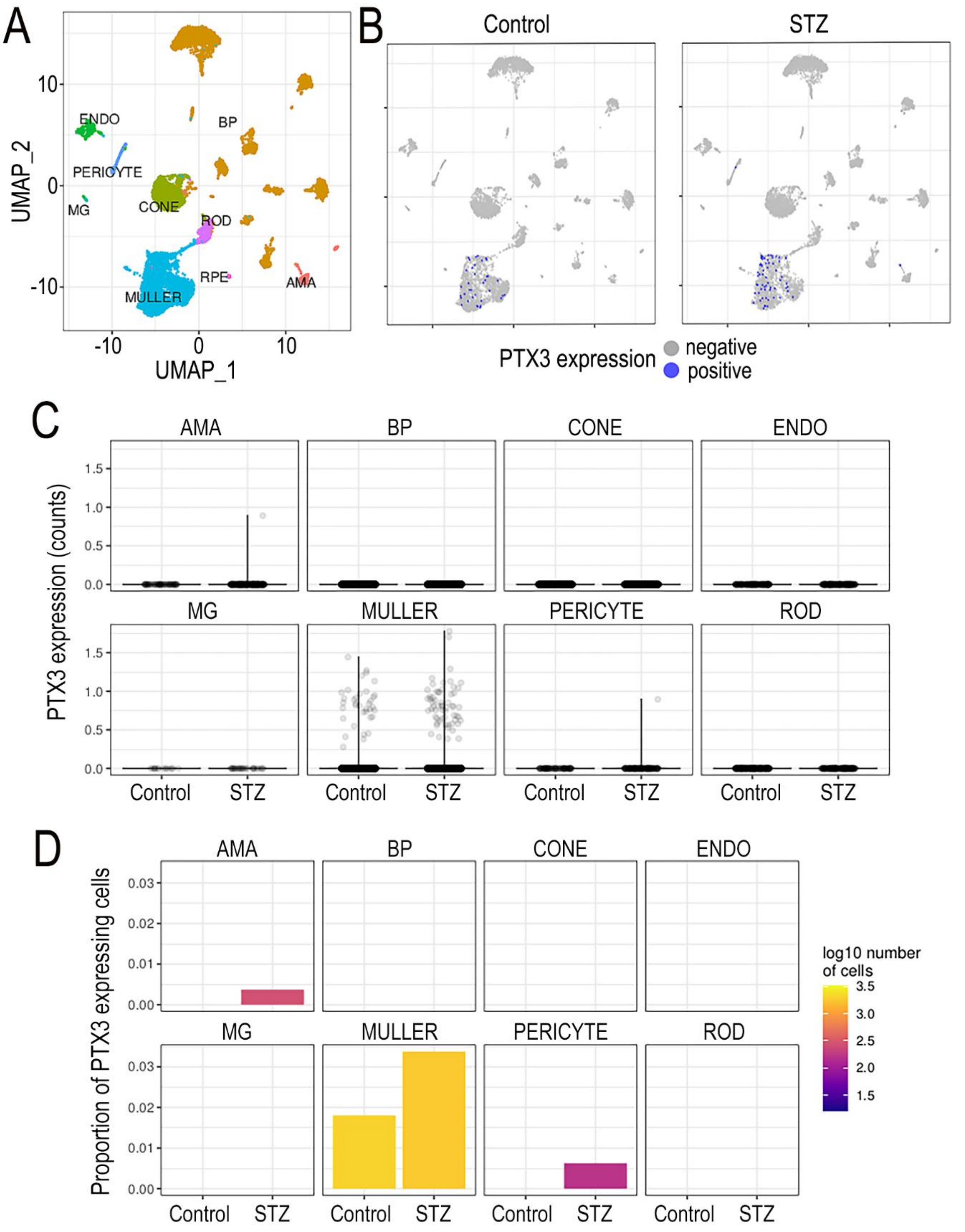

Fig. S4. scRNAseq analysis of retinal tissue from the STZ mouse diabetic model identifies Muller cells as the retinal cell type with the highest PTX3 expression. (A) UMAP clustering of cells with annotation for 9 retinal cell types. (B) UMAP visualization of PTX3 expression. Cells expressing PTX3 are shown in light blue (positive), and not expressing cells are shown in grey (negative). (C) Dot plots to show PTX3 expression as counts per cell across the distinct retinal cell types. (D) Bar plots to depict the proportion of cell types that express PTX3. The absolute number of cells sequenced is shown by the color scale.

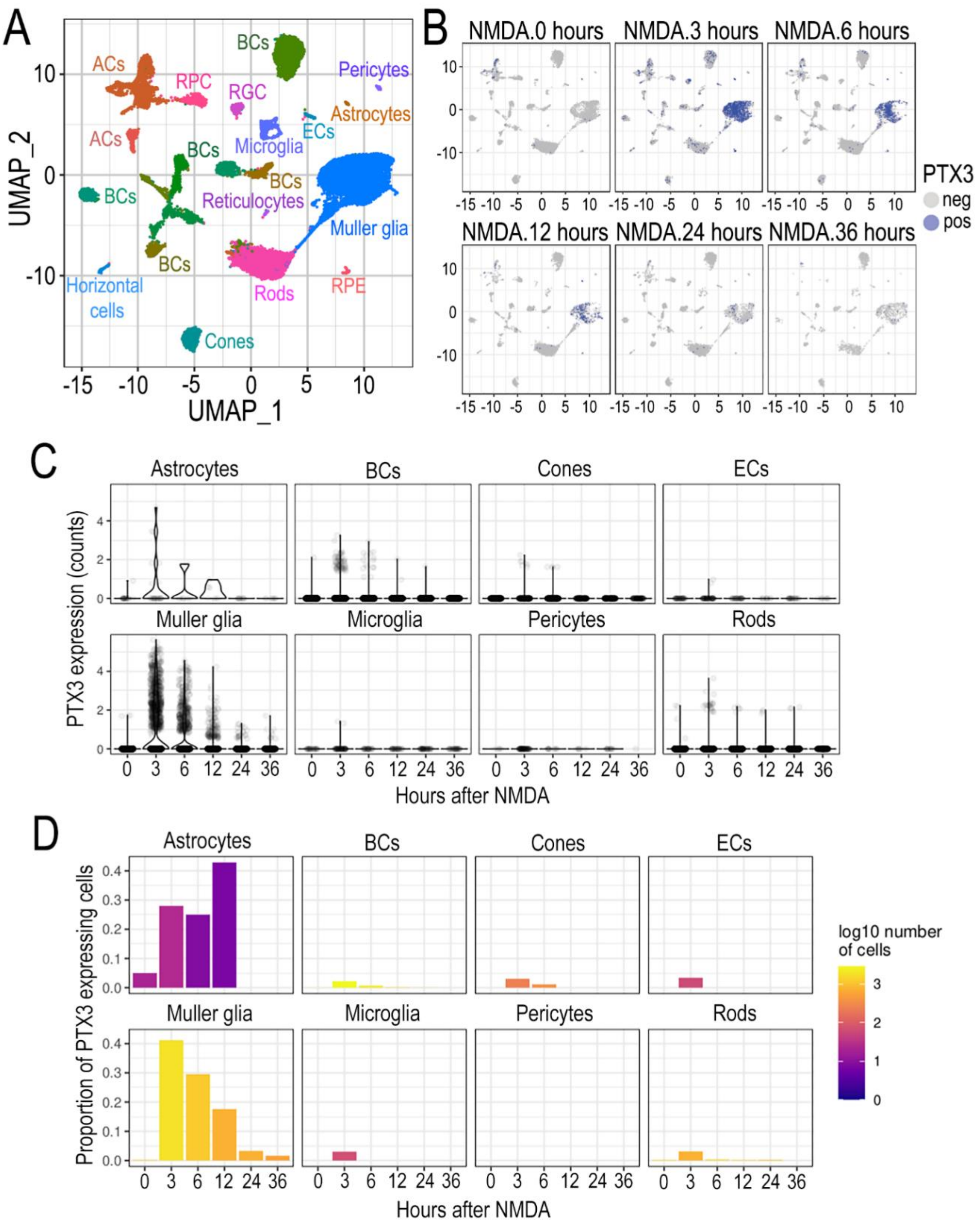

Fig. S5. scRNAseq analysis of the NMDA retinal injury model for PTX3 expression. (A) UMAP clustering of cells with annotation for 14 retinal cell types. (B) UMAP visualization of PTX3 expression after NMDA injury from 0 up to 36 hours. Cells expressing PTX3 are shown in light blue (pos: positive), and not expressing cells are shown in grey (neg: negative). (C) Violin plots to depict PTX3 expression as counts per retinal cell type across the timeline. (D) Bar plots to show the proportion of cells expressing PTX3 per cell type across the time course. Absolute number of cells is shown by the color scale.

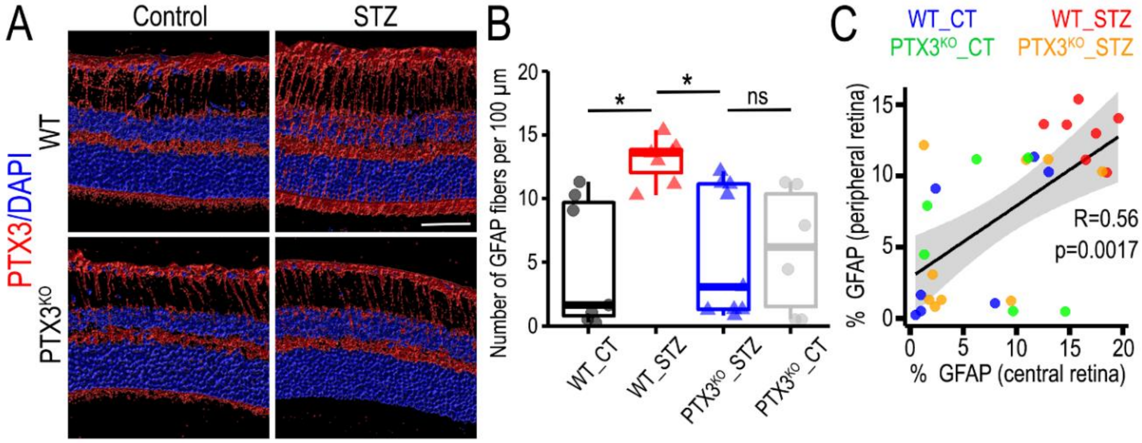

Fig. S6. PTX3 deficiency reduced GFAP expression in diabetic peripheral retinas.
(A) Immunofluorescence for GFAP (red) in peripheral retinal cross sections from 9-month diabetic and non-diabetic mice, wildtype (WT) and PTX3^{KO}. Scale bar: 50 µm. (B) Quantification of GFAP staining as number of fibers per 100 µm, n = 5-8, *p<0.05. (C) Pearson correlation coefficient of GFAP percentage between central and peripheral retina. CT: control vehicle, STZ: streptozotocin.

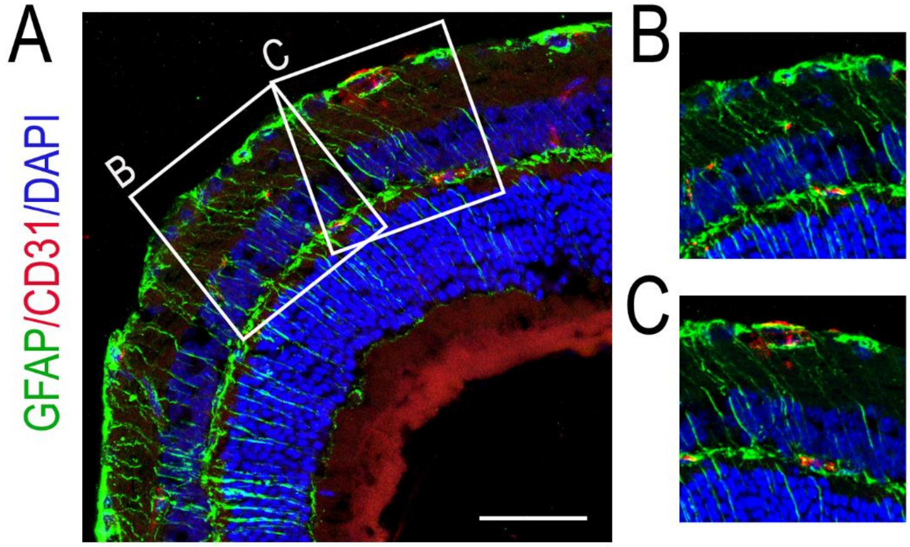

Fig. S7. PTX3 staining profile in mouse retinal explants treated with TNFα.
(A) Immunofluorescence for GFAP (green) and CD31 (red) in peripheral retinal cross sections from retinal explants exposed to TNFα. Scale bar: 50 μm. (B, C) Higher magnification images for insets shown in A.

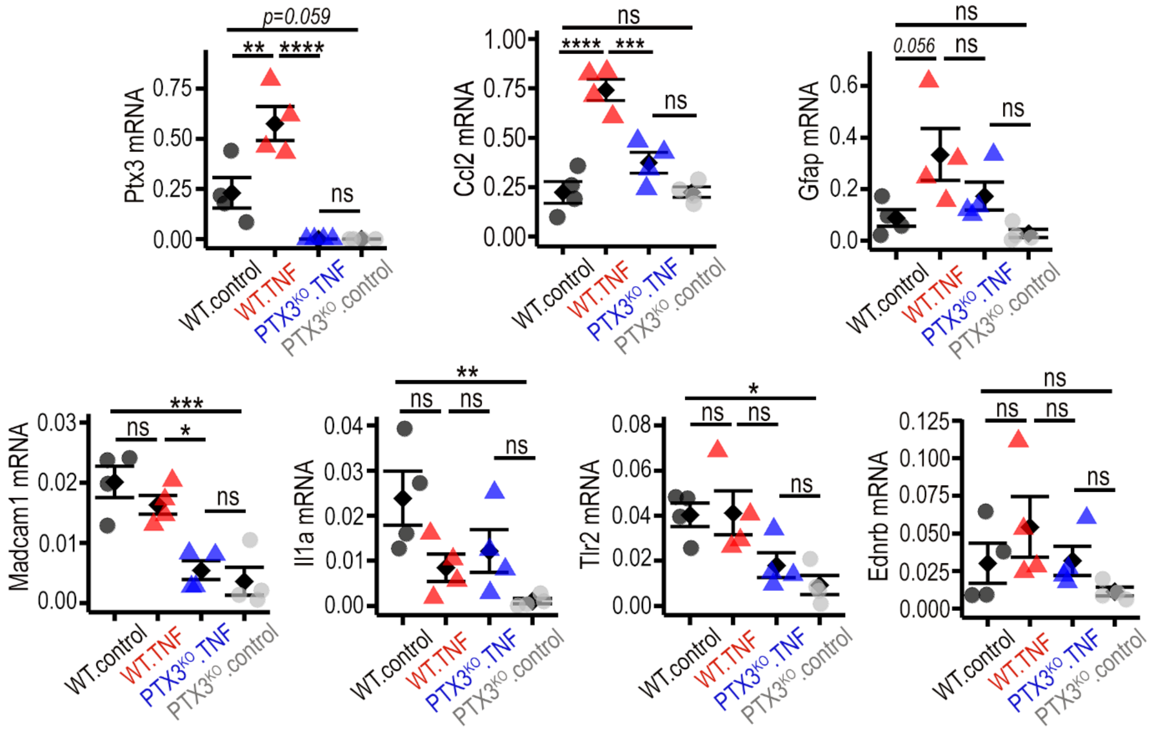

Fig. S8. Evaluation of gene expression changes in mouse retinal explants treated with TNFα. RT-qPCR was performed on total RNA from whole retinas and relative expression evaluated in relation to housekeeping gene RPL11. n = 4, ns = not significant, *p<0.05, **p<0.01, ***p<0.001, ****p<0.0001.

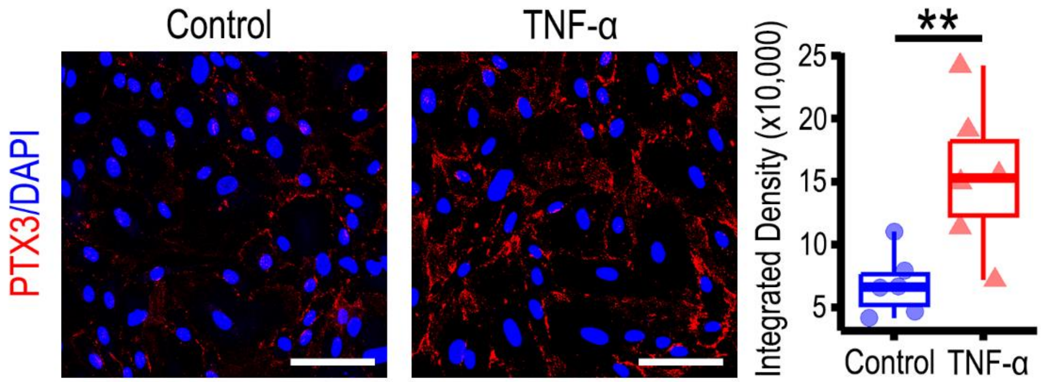

Fig. S9. PTX3 expression is increased in human retinal astrocytes treated with TNF-α. Immunocytochemistry of human retinal astrocytes for PTX3 (red) comparing control (n=6) and TNF-α treated cells (n = 6). Nuclei are stained with DAPI (blue). Quantification of GFAP staining was performed using integrated density. **p<0.01. Scale bar: 100 μm.

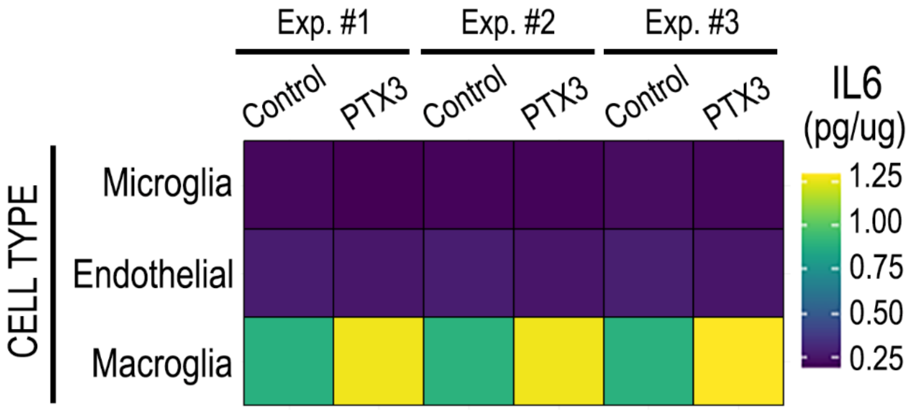

Fig. S10. Cell type screening for response to recombinant human PTX3. Human microglia, endothelial microvascular cells and astrocytes (macroglia) were treated with vehicle or recombinant human PTX3 in three independent experiments. Conditioned media was collected for IL6 measurements using ELISA. Results shown as a heatmap for secreted IL-6 levels across the three cell types.

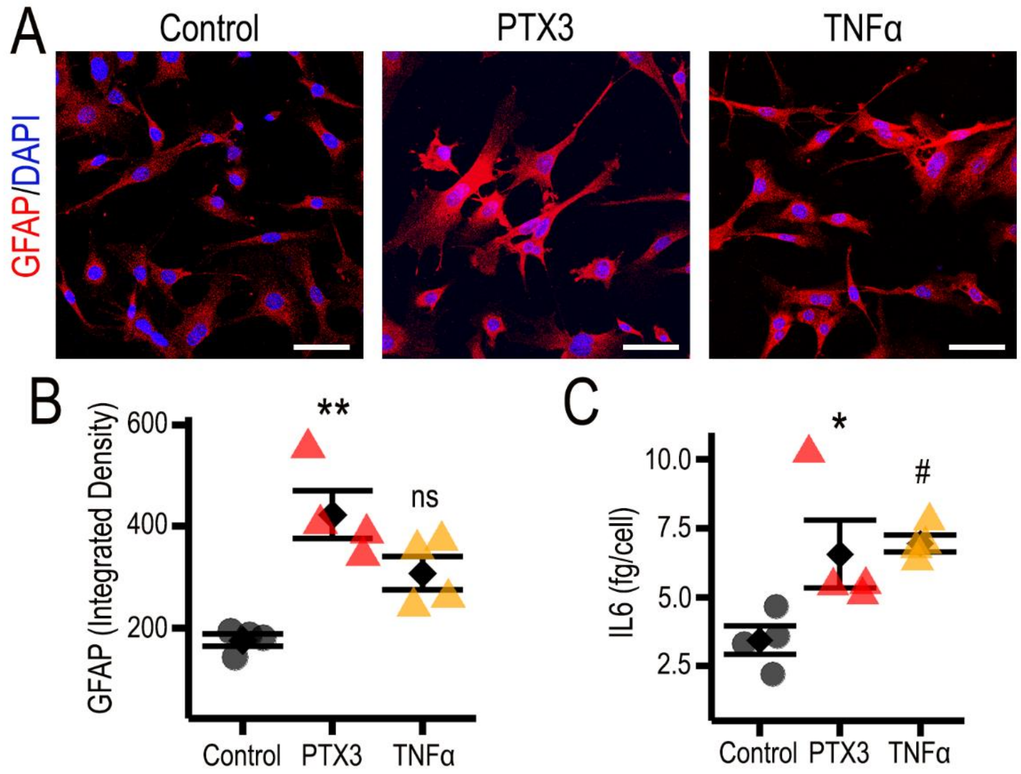

Fig. S11. GFAP expression is increased in mouse retinal glia treated with PTX3.
(A) Immunocytochemistry for GFAP in db/db mouse retina-derived mixed glia cultures treated with PTX3 or TNF-α. PTX3 shown in red and nuclei stained with DAPI in blue. Scale bar: 50 μm. (B) Dot plot depicting quantification of GFAP signal as integrated density, to compare vehicle-treated controls with PTX3- or TNF-α-treated cells, n = 4, ns: not significant, *p<0.05. (C) Quantification of IL6 released to culture media by ELISA and normalized to cell number, n = 4, *p<0.05 for control vs. PTX3, #p<0.05 for control vs. TNF-α.

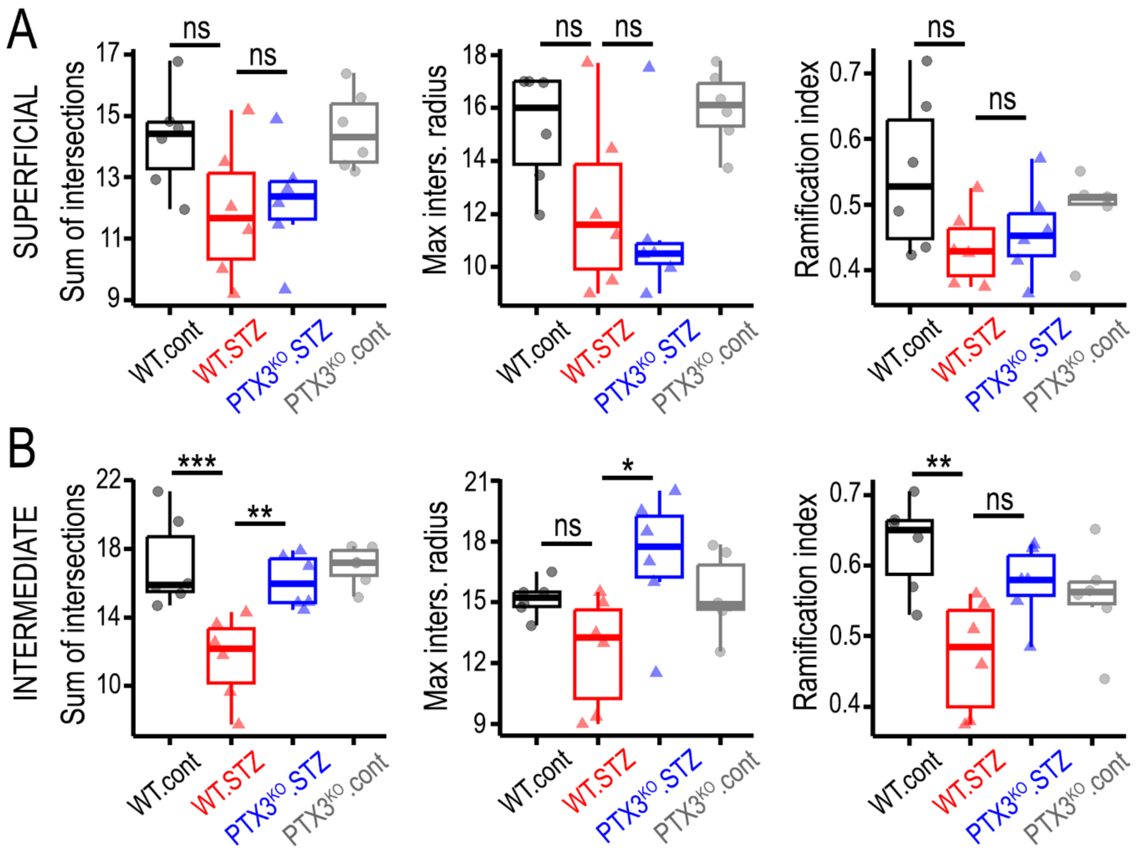

Fig. S12. Sholl analysis readouts for retinal microglia in the superficial or intermediate retinal layers. Sum of intersections, maximum intersection radius and ramification index were measured at the superficial (A) and intermediate (B) retinal layers, n = 5-6. *p<0.05, **p<0.01, ***p<0.001, ns: not significant.

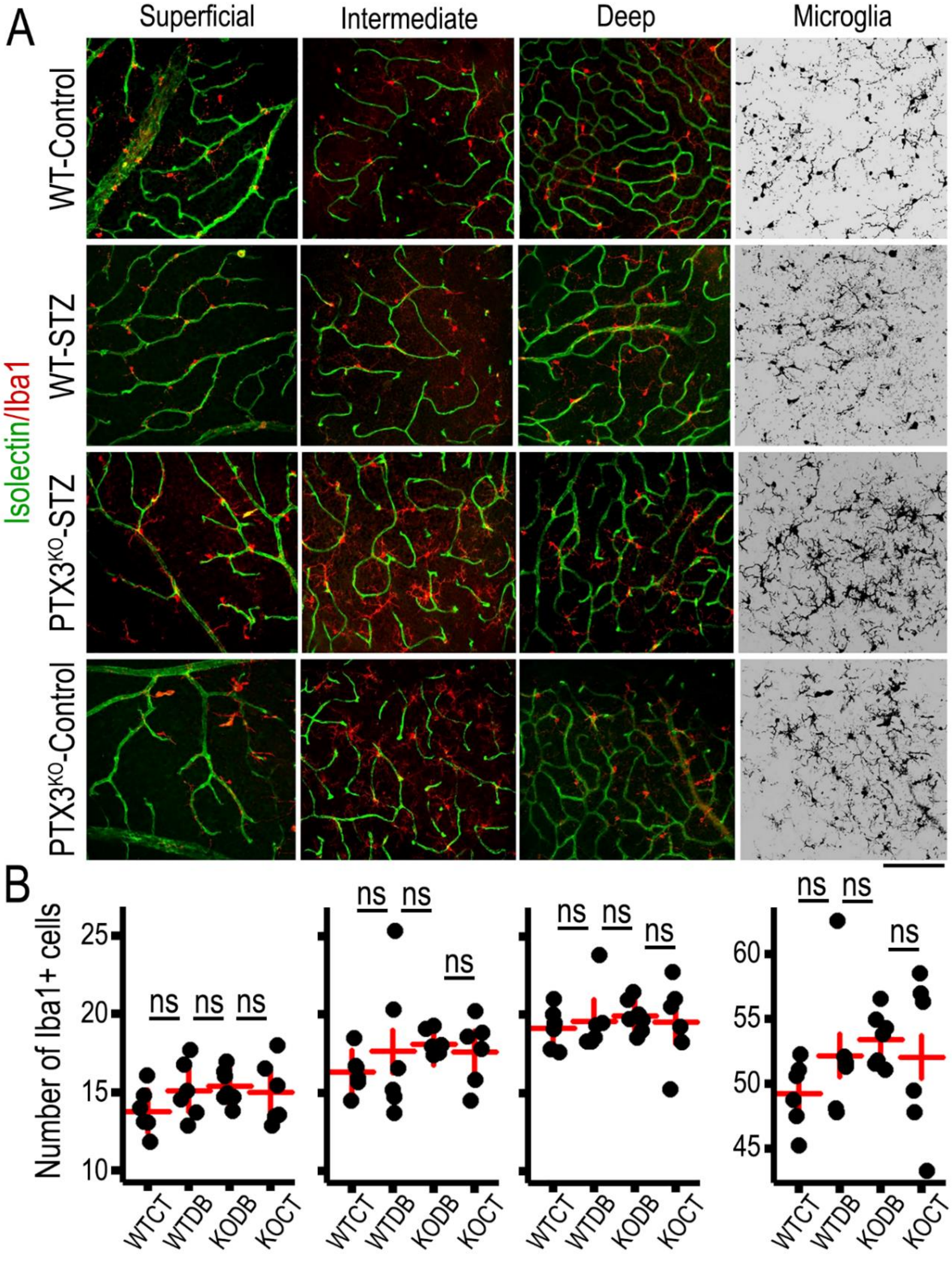

Fig. S13. Quantification of microglia numbers at the superficial, intermediate, and deep retinal layers. (A) Confocal microscopy images showing Isolectin fluorescent staining for retinal vessels (green) and Iba1+ microglia (red) in the three retinal layers. Merged layers for Iba1 staining are shown in black and white images. Scale bar: 50 µm. (B) Quantification of Iba1+ cells across the three retinal layers and totals, n = 5-7, ns: non-significant.

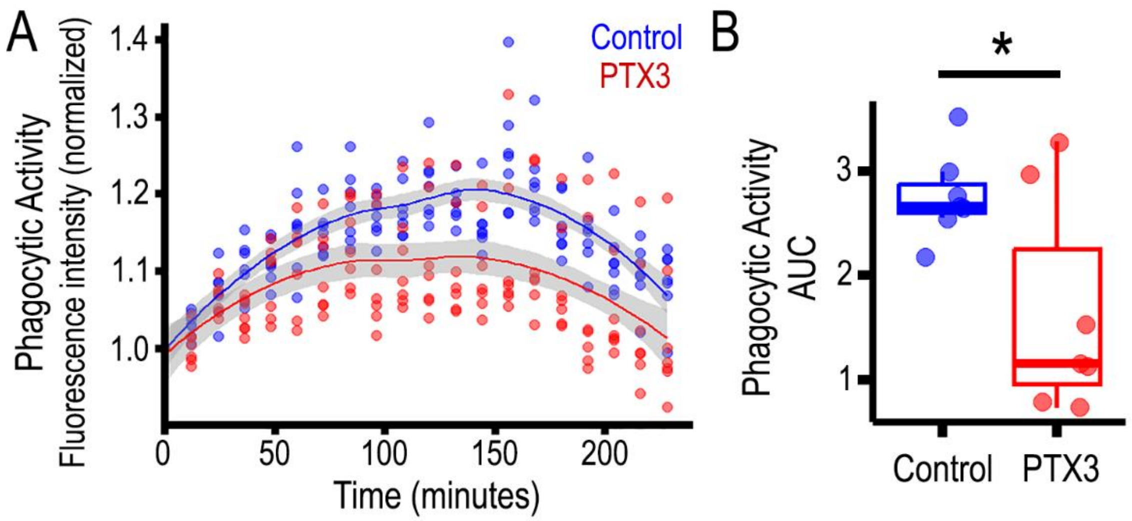

Fig. S14. PTX3 diminished phagocytic capacity of human microglia. Human microglia cells were treated with recombinant human PTX3 or vehicle for 16 hours and then exposed to pHRODO-SE tagged apoptotic bodies. Live cell fluorescent images were taken over a period of 240 min and fluorescence intensity plotted as a readout of phagocytosis. *p<0.05.

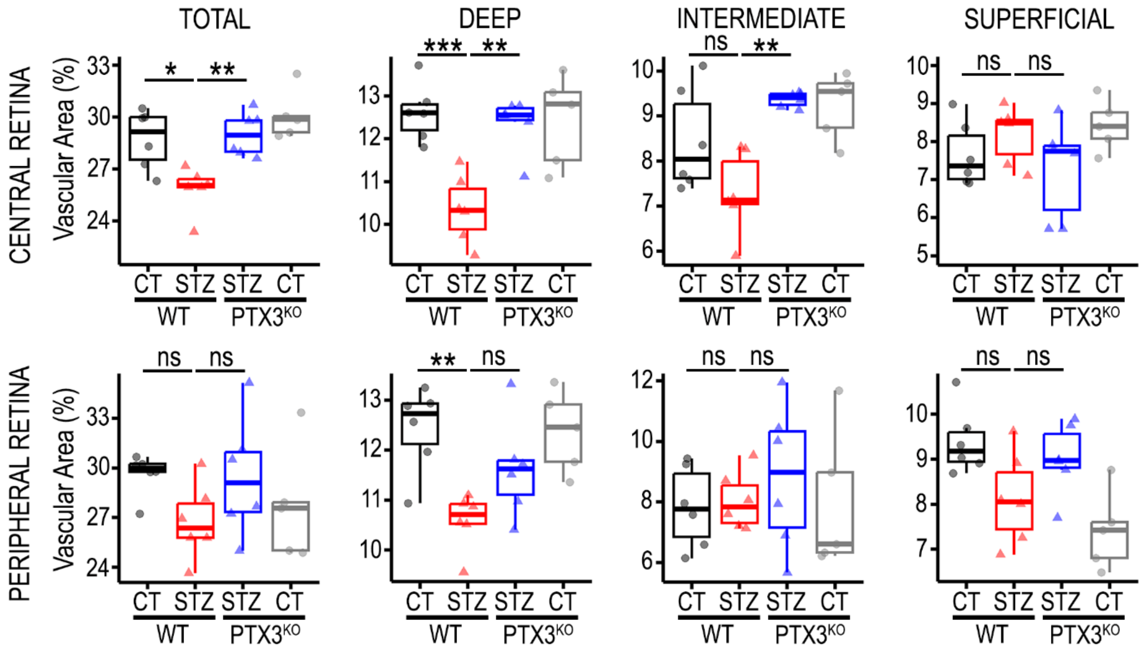

Fig. S15. Vascular densities at different retinal layers in wild type and PTX3^{KO} mouse, diabetics and controls. Fluorescent confocal scanning microscopy images were analyzed using Angiotool platform to measure total vascular area (%) and vascular area across the superficial, intermediate, and deep retinal layers. Top figure shows quantification results for central retina, bottom figure shows peripheral retina results. n = 5-6 *p<0.05, **p<0.01, ***p<0.001, ns: non-significant.

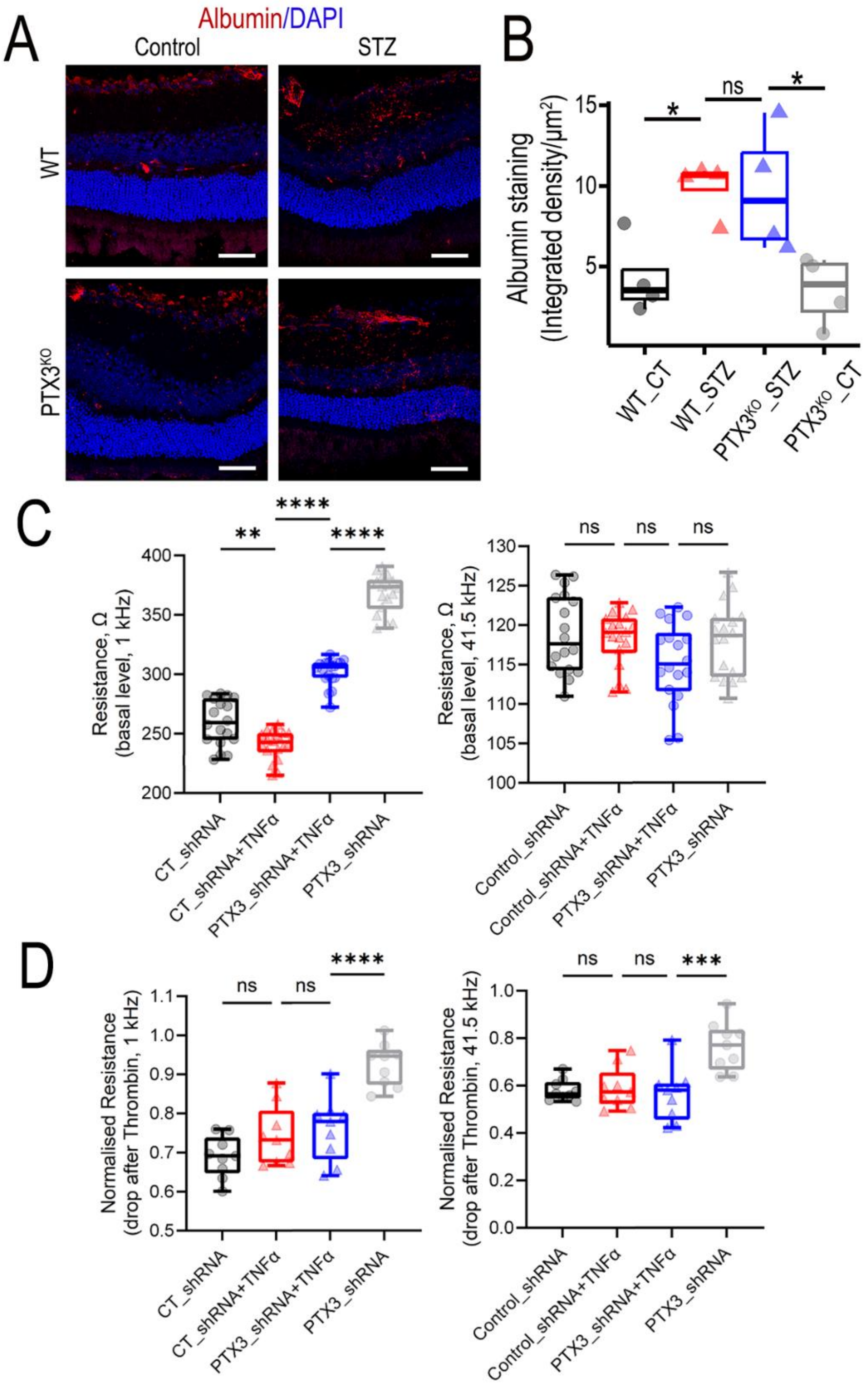

Fig. S16. Blood retinal barrier breakdown induced by diabetes and PTX3. (A) Microscopy images showing Albumin staining within retina in red as a marker of vascular leakage. Nuclei counterstained with DAPI in blue. Scale bar: 50 µm. (B) Quantification of albumin staining by integrated density per µm^2 of retinal tissue, n = 4, *p<0.05, ns: non-significant. (C) Human endothelial cells with knockdown of PTX3 or controls were treated with TNFα and the transendothelial electrical resistance was measured using the Maestro system at 1 and 41.5 kHz. n = 18, **p<0.01, ****p<0.0001, ns: not significant. (D) Endothelial cells were exposed to thrombin to disrupt the endothelial barrier. Impedance was measured and normalized to time of thrombin addition. n = 9, ***p<0.001, ****p<0.0001, ns: not significant.

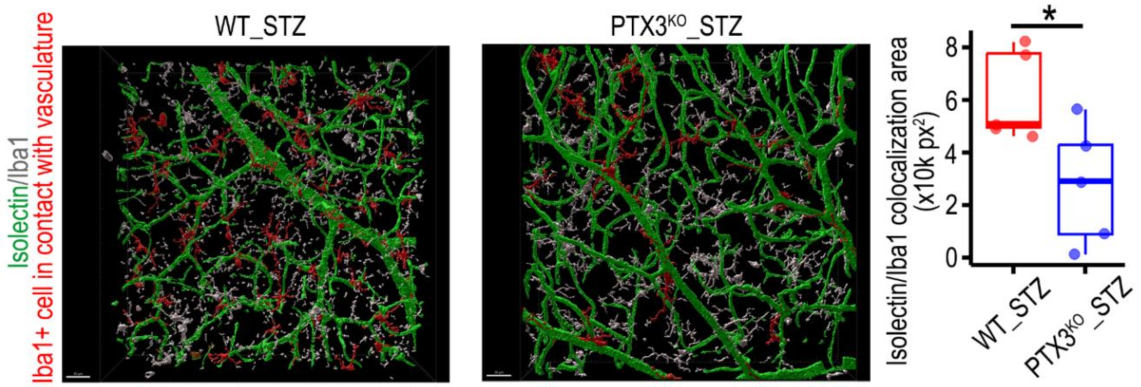

Fig. S17. Mouse diabetic retinas lacking PTX3 exhibited decreased Iba1+ microglia-vascular interaction. Confocal z-stacks were used to generate 3D surfaces for lectin+ vasculature (green) and Iba1+ microglia (red/white) using Imaris software. The Surface-Surface Coloc Imaris Matlab Xtension was applied on the 3D rendered surfaces, enabling the detection of contact points between lectin+ and Iba1+ signals. Microglia in direct contact with lectin+ vessels are shown in red, other microglia shown in white. Quantification of lectin/Iba1+ 3D surface colocalization area in diabetic wild type and $PTX3^{KO}$ mice, n = 4-5. *p<0.05. Scale bar: 30 μm.

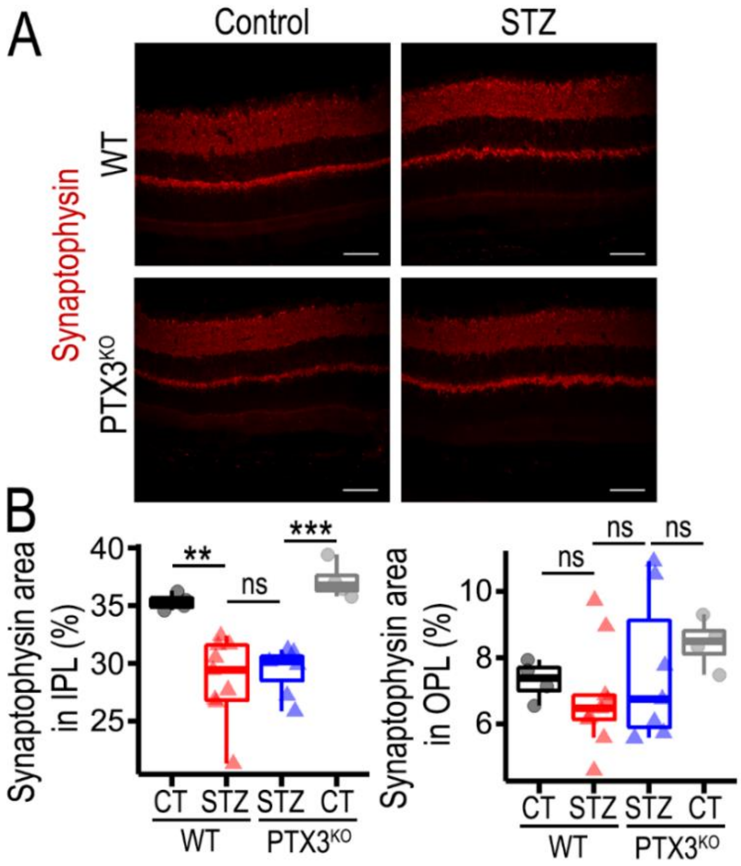

Fig. S18. Diabetes-induced changes in synaptophysin staining were similar in PTX3^{KO} and WT mouse retinas.

(A) Immunohistochemistry staining of retinas for Synaptophysin (red) in 9-month PTX3^{KO} and WT diabetic (STZ) mice and age-matched controls. (B) Quantification as percentage of synaptophysin staining area in the IPL and OPL. **p<0.01, ***p<0.001. IPL=inner plexiform layer, OPL=outer plexiform layer, ns=non-significant. Scale bar: 50 μm.

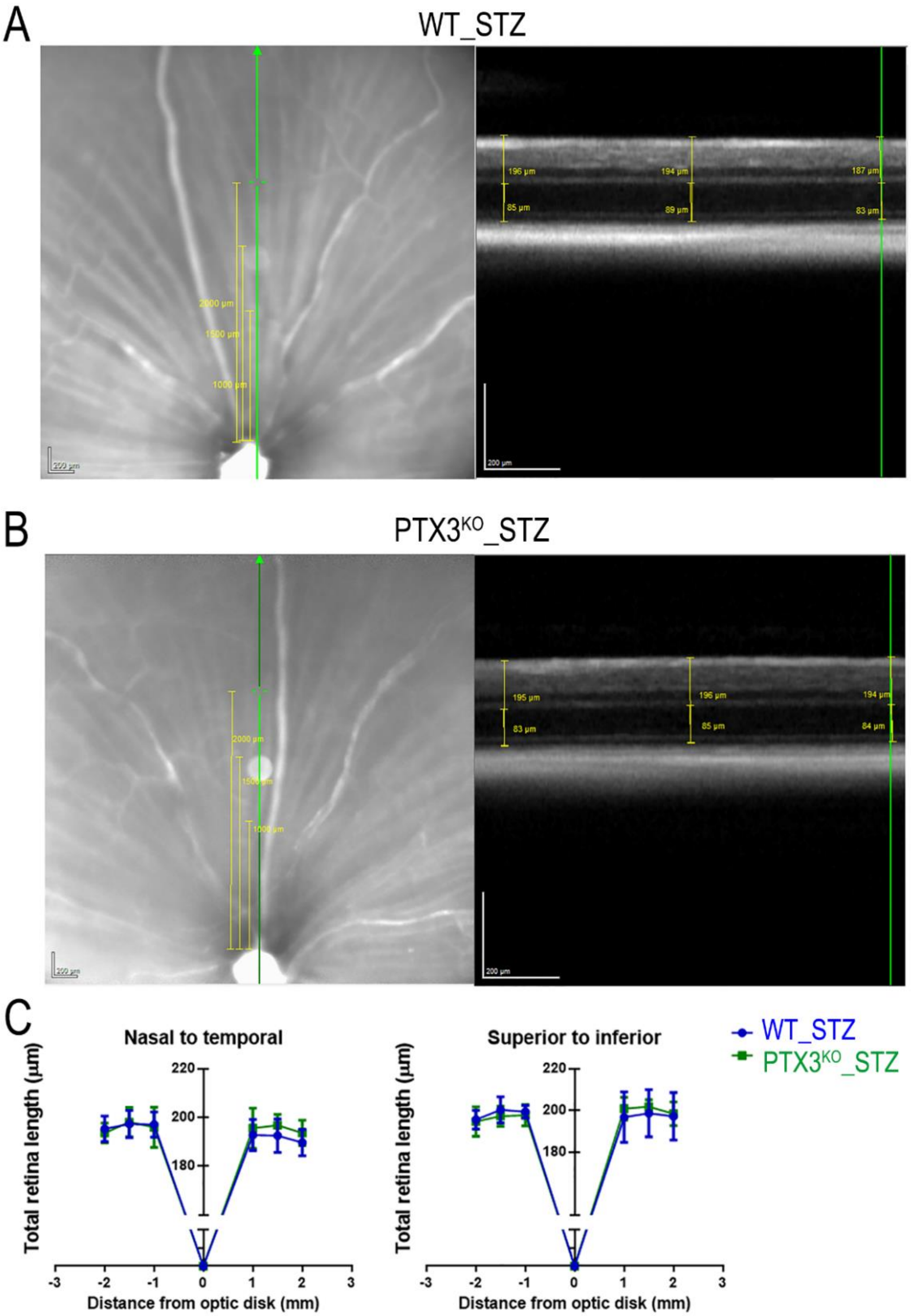

Fig. S19. Optical Coherence Tomography (OCT) retinal thickness evaluation did not show differences between diabetic wildtype and PTX3^{KO} retinas. OCT scanning images were taken in diabetic (9-month STZ) mice. Representative images shown for WT (n = 5) (A), and PTX3^{KO} (B) mice (n = 7). Scale bar: 200 µm. The green lines show the retinal area examined. Yellow bars represent the length of the retinal areas and layers measured. (C) Quantification of retinal length, when retina was examined horizontally (nasal to temporal) and vertically (superior to inferior). No differences were found when comparing PTX3^{KO} (green line) and WT (blue line).

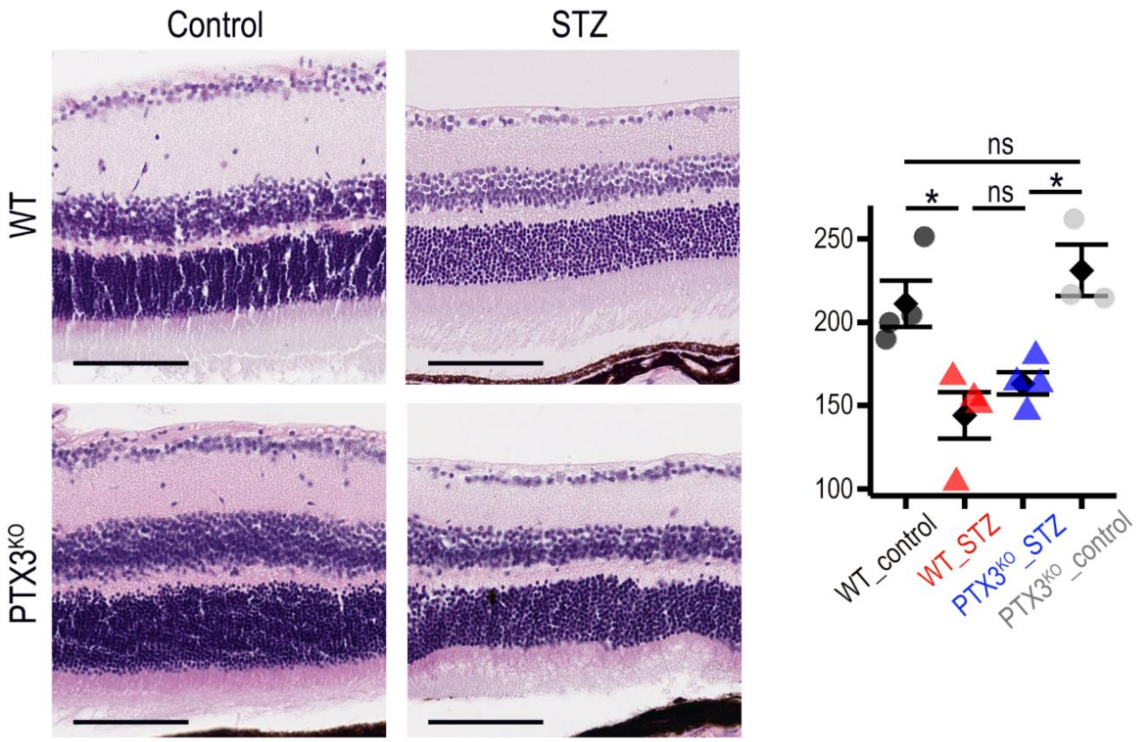

Fig. S20. Retinal thickness evaluation by H&E stain. Representative images for cross sections from central retina stained for H&E after 9 months of diabetes induction, compared to non-diabetic controls. Scale bar: 200 μm. Retinal thickness was measured from the outer nuclear layer to the inner limiting membrane, and results shown in dot plot, n = 4, ns: not significant, *p<0.05.

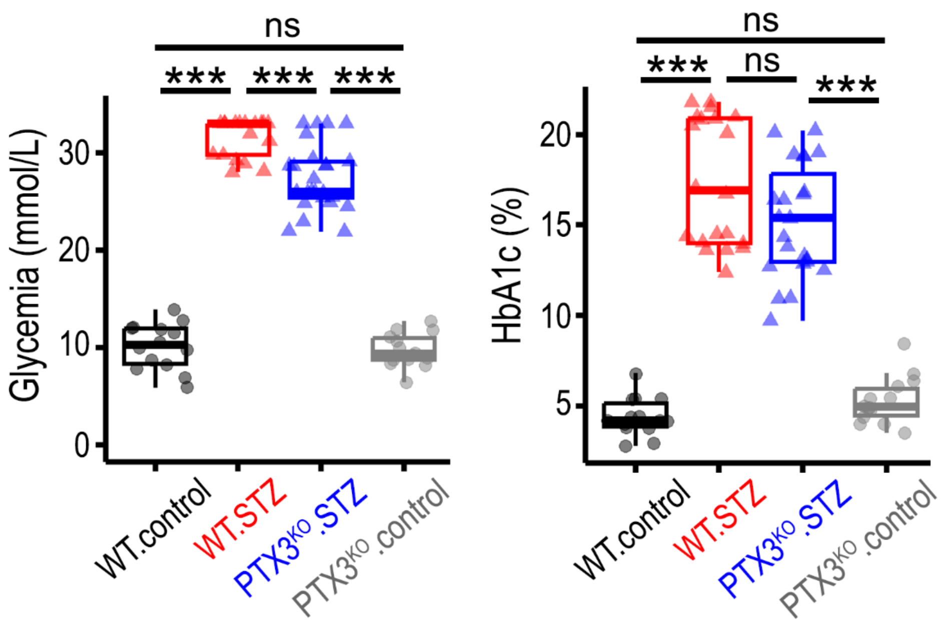

Fig. S21. Supplementary Figure 21. The PTX3 knockout genotype did not affect HbA1c levels in diabetic mice. Evaluation of glycemia and HbA1c in mouse bloods after 9 months of diabetes induction. ***p<0.001, ns: not significant.

Table S1. Details for antibodies used

Antibody	Host	Manufacturer and catalog number	Dilution
Pentraxin 3 polyclonal	Rabbit	Manufactured by Humanitas Research Hospital, against recombinant human PTX3	1:50
GFAP monoclonal	Mouse	ThermoFischer Scientific; MA5-12023	1:100
GFAP polyclonal	Rabbit	Dako; Z0334	1:200
Il1β polyclonal	Rabbit	Abcam; Ab9722	1:100
Iba1 monoclonal	Rabbit	Abcam; Ab178846	1:100
Collagen 4A1 polyclonal	Rabbit	Origene; BP5031	1:100
Cone Arrestin polyclonal	Rabbit	Merck; Ab15282	1:1000
Brn3a monoclonal	Rabbit	Abcam; Ab245230	1:100
Synaptophysin	Rabbit	Abcam; Ab14692	1:200
Pentraxin 3	Goat	R&D Systems; AF2166	1:100
Aggrecan	Mouse	Abcam; Ab3778	1:200
Tenascin-C	Mouse	Abcam; Ab88280	1:100
Versican	Mouse	Thermo; MA5-27638	1:100
Endostatin	Rabbit	Thermo; PA1-601	1:200
Hyaluronic Acid Binding Protein – biotinylated	Source: Bovine nasal cartilage	Amsbio; 400763	10 µg/ml

Movie S1 (separate file). Recombinant PTX3 diminishes phagocytosis activity in human microglia. Time-lapse live cell imaging of human microglia HMC3 cocultured with pHrodo succinimidyl ester red apoptotic bodies. Apoptotic bodies were generated from human retinal microvascular endothelial cells and human retinal astrocytes treated with Staurosporine. Images were taken using a TIRF microscope every 12 minutes up to 4 hours. Red signal indicates intracellular accumulation of apoptotic bodies within microglia cells.
